# Platelet-derived microparticles increase the interaction of colorectal cancer cells with the endothelium to promote metastatic events

**DOI:** 10.1186/s12967-025-06858-9

**Published:** 2025-07-25

**Authors:** Izabela Papiewska-Pająk, Hassan Kassassir, Wiktoria Moczkowska, Marcin Braun, Anna Rybicka, Joanna Boncela, M. Anna Kowalska

**Affiliations:** 1 Laboratory of Cellular Signaling, Institute of Medical Biology, Polish Academy of Sciences, Lodz, Poland; 2 Department of Pathology, Medical University of Lodz, Lodz, Poland; 3 Department of Hematology, The Children’s Hospital of Philadelphia, Philadelphia, PA USA; 4 BioMedChem Doctoral School of the UL and Institutes of the PAS, University of Lodz, Lodz, Poland

**Keywords:** Platelet-derived microparticles, Colorectal cancer, Endothelial cells, Metastasis, Intravasation, Extravasation

## Abstract

**Background:**

The major challenge in colorectal cancer (CRC) therapy involves the formation of distant metastases, which represent the primary cause of treatment failure and patient death. To undergo metastasis, cancer cells of an invasive phenotype must intravasate and extravasate the blood or lymphatic vessels to reach distant sites. Platelet-derived microparticles (PMP) are considered important factors in various diseases, including CRC. Here, we examined the influence of PMP on the intravasation and extravasation abilities of CRC cells and established associations with these PMP to metastatic events in vivo.

**Methods:**

Fluorescence microscopy was used to investigate the effects of PMP on CRC cell adhesion to the endothelial (HMEC-1) monolayer, endothelial integrity and CRC cell transendothelial migration. Quantitative real-time PCR and flow cytometry were used to assess endothelial gap junction and tight junction protein expression. For the in vivo experiments we performed intrasplenic injections of CRC cell lines with different molecular characteristics into immunodeficient mice, followed by the intravenous administration of multiple human PMP. The presence of metastases and inflammation in the liver was confirmed via histopathological examinations. Immunohistochemical analyses of human CD41 in metastatic lesions were performed to detect human PMP. Platelet surface activation markers and the plasma concentrations of inflammatory cytokines were evaluated via flow cytometry. The plasma levels of metalloproteases (MMPs) 2 and 9 were measured via ELISA.

**Results:**

Our study revealed that PMP enhanced CRC cell adhesion to endothelial cells and transendothelial migration. PMP injections increased the number of metastases in the liver and the concentrations of total MMP-2 and human MMP-9 in the plasma of mice injected with selected CRC cell lines. PMP were observed to be present at the margins of metastatic lesions and endothelial capillaries. PMP injections also increased the level of platelet receptors, which determine blood platelet activation and reactivity.

**Conclusions:**

Our in vitro findings suggest that PMP can promote CRC cell adhesion to endothelial cells, which contributes to cancer cell extravasation. PMP can also disrupt the integrity of endothelial cell junctions and enhance the transendothelial migration of CRC cells. PMP demonstrate a supportive role in metastatic events via the upregulation of plasma levels of metalloproteases.

**Supplementary Information:**

The online version contains supplementary material available at 10.1186/s12967-025-06858-9.

## Introduction

The effect of circulating blood platelets (PLT) on metastasis in various cancers is generally well known; however the influence of platelet-derived microparticles (PMP), which are released from activated platelets, is still poorly understood. Thus, we focused our research on the role of PMP in colorectal cancer (CRC), as the occurrence of metastases in this type of cancer rapidly reduces the 5-year survival rate of CRC patients [1]. When considering that metastatic seeding often precedes diagnosis, further efforts are needed to understand the mechanisms of metastatic events.

To form metastatic lesions, several stages of metastatic events are necessary, including the intravasation of cancer cells, their survival in the circulation, extravasation, and the colonisation of premetastatic niches [2]. To enter or leave the circulation, after adhering to the endothelium, cancer cells must pass through the endothelial cell (EC) layer in blood vessels and undergo migration. The rate at which tumour cells gain access to the peripheral circulation and subsequently undergo extravasation can affect the efficiency of metastasis; therefore the activities of the factors that can influence the process of intravasation and extravasation should be thoroughly investigated [3]. The participation of specific factors that are necessary for cancer cell-endothelium transmigration has been extensively investigated [4]; however, the involvement of PMP, in this process remains to be clarified.

PMP are enriched in bioactive contents such as proteins (e.g. signalling molecules and membrane receptors), nucleic acids or lipids, and these molecules can modify the phenotype and function of recipient cells [5]. PMP infiltration has been observed in multiple solid tumour types; however, it has not been observed in unaffected normal tissues [6]. Due to the small diameter of endothelial capillaries, disturbed blood flow, and increased permeability of the tumour vasculature, cancer cells are more accessible to PMP than to PLT. Our research team, along with other researchers, have proposed the notion that PMP can interact with cancer cells and regulate cancer spread by supporting cell invasion [7–9]. Moreover, cancer cells can stimulate platelets in the circulation of patients, thereby leading to the release of PMP which can subsequently be incorporated by cancer cells to potentiate their invasive phenotype [10]. Moreover, transfusions after surgery and post-surgery therapies can allow for the delivery of platelets from healthy donors, which can be partially activated and serve as an additional source of PMP in the circulation.

Our previous findings have suggested that PMP (when incorporated into CRC cells) can stimulate the expression and activity of the metalloproteases MMP-2 and MMP-9, which leads to a more invasive cell phenotype in vitro [7]. The activity of the gelatinases MMP-2 and MMP-9 is crucial for passing through the extracellular matrix, which is the first barrier that cells with an invasive phenotype must overcome prior to the intravasation process [11]. Such increased activity has been demonstrated in CRC in vitro [7] and in colorectal cancer patients [12]. These observations prompted us to further investigate this phenomenon. In this study, we investigated the influence of PMP on the integrity of the endothelial cell layer in vitro, as this experimental approach reflects intravasation and extravasation events that occur during metastasis. Additionally, to evaluate the role of PMP in metastatic events in CRC, we used a mouse model of intrasplenic injections of CRC cell lines, followed by the intravenous administration of PMP.

## Methods

**Scheme 1:**
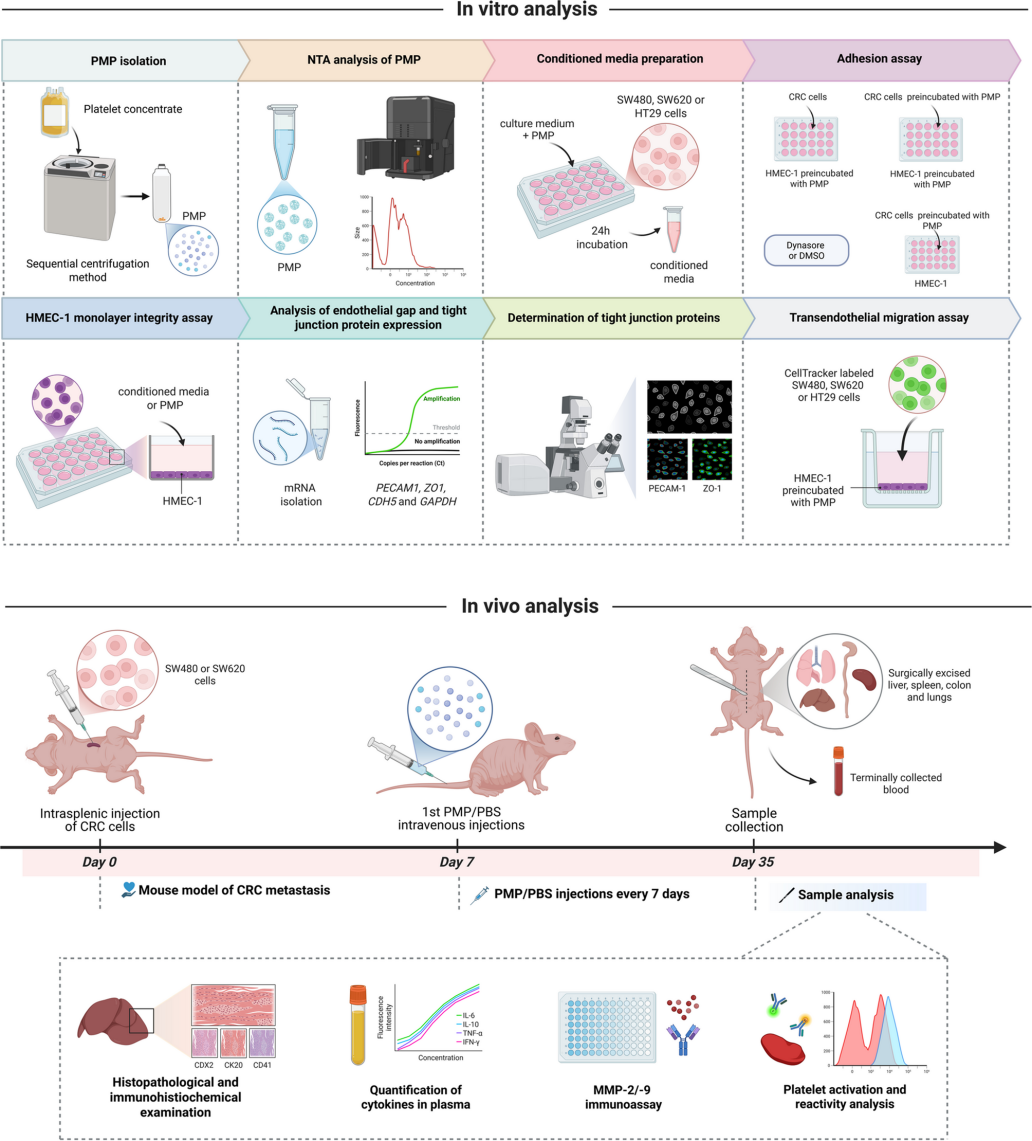
A schematic diagram of the procedures used in this study. Created in BioRender. Moczkowska, W. (2025) https://BioRender.com/84l2ad1

The details of the cell lines, PMP isolation methods, in vitro experiments and animal experiments are listed in the Supplementary Materials and methods. The in vitro an in vivo procedures used in this study are graphically presented in the Scheme 1.

### Cell culture

Human colorectal adenocarcinoma cell lines with different phenotypic migratory potentials, including HT29 (epithelial, CMS3), SW480 (mesenchymal, CMS4) and SW620 (strongly mesenchymal, CMS4) as well as the endothelial HMEC-1 cell line (immortalised dermal microvascular endothelial cells) (ATCC CRL-3243 ™), were purchased from the American Type Culture Collection (ATCC, Manassas, VA, USA). HT29 cells were cultured in McCoy’s 5 A medium (Thermo Fisher Scientific, Waltham, MA, USA) and SW480 and SW620 cells were cultured in RPMI 1640 medium (with the ATCC modification, Thermo Fisher Scientific). The HMEC-1 cell line was cultured in MCDB131 medium supplemented with the following growth factors: 10 ng/mL epidermal growth factor, 1 µg/mL hydrocortisone, and 10 mM glutamine. Unless otherwise stated, all of the experiments were performed in FBS-free media (experimental media). For the experimental studies, CRC cells between the 10th and 20th passages and HMEC-1 cells between the 3rd and 9th passages were used.

### Isolation and characteristics of platelet-derived microparticles (PMP)

PMP were obtained from platelet concentrates purchased from the Regional Centre of Blood Donation and Blood Treatment in Łódź in accordance with applicable law. First, the platelets were separated from the plasma via centrifugation at 200 × g for 20 min at RT. After two washes with Tyrode’s buffer, the platelets were stimulated with 2 U/mL thrombin and 2.5 mM CaCl_2_ for 20 min on a rotary shaker. PMP were obtained after centrifugation of stimulated platelets at 1,500 × g for 20 min at RT followed by ultracentrifugation of the resulting suspension at 100,000 × g for 2 h at 4 °C, as described in detail in a previous study [13]. Before starting the experiments, we performed NTA, which is characteristic of the sizes of PMP (Fig. S1).

### Preparation of conditioned media after CRC cell stimulation with PMP

CRC cells were seeded in the 24-well plates for 48 h. At 80–90% confluence, the medium was replaced with experimental medium, and PMP were added (50 µg of PMP/10^6^ cells for a final concentration of 100 µg/ml) for 24 h. Subsequently, the conditioned medium (obtained from PMP–stimulated CRC cells and from nonstimulated CRC cells [as a control]) was collected, and centrifuged at 180 × g for 5 min at RT to remove cellular debris; afterwards the supernatant was collected and frozen at -20 °C until use.

### CRC cell adhesion to the endothelial cell layer

HMEC-1 (4 × 10^5^) were seeded in the 24-well plates for 48 h. At 80–90% confluence, the full medium was changed to medium without EGF and hydrocortisone for 16 h before the experiment began. CRC cells alone, HMEC-1 cells alone or a combination of both CRC cells and HMEC-1 cells were preincubated with PMP (50 µg of PMP/10^6^ cells for a final concentration of 100 µg/mL) and with or without addition of dynamine inhibitor, Dynasore (final concentration of 50 µM) or DMSO (final concentration of 0.1%, control) for 4 h in appropriate experimental medium. Ultimately, the adhesion of CRC cells (2 × 10^5^) labelled with CellTracker™ Green CMFDA dye was determined using a fluorescence inverted microscope.

### Assessment of the integrity of the HMEC-1 monolayer after conditioned medium or PMP treatment

HMEC-1 cells (4 × 10^5^) were seeded in the 24-well plates for 48 h. At 80–90% confluence, the full medium was changed to the medium without EGF and hydrocortisone for 16 h before the experiment began. The conditioned media of previously PMP-treated CRC cells (final concentration of 100 µg/mL) or PMP alone (final concentration of 100 µg/mL) were added to HMEC-1 cells for 4 h, and the integrity of the HMEC-1 monolayer (which was previously labelled with CellTracker™ Green CMFDA dye) was determined. The areas covered with fluorescently labelled HMEC-1 cells were calculated via ImageJ software (Fiji).

A quantitative barrier assay was used as previously described in [14] (with some modifications). Briefly, HMEC-1 cells (4 × 10^5^) were seeded in full medium on 0.4 μm transwell inserts in the 24-well plate for 3 h, after which the cells were treated with conditioned media or PMP as described above. Dextran conjugated with fluorescein isothiocyanate (FITC) (1 µg/mL) was added to the upper chamber and the amount of diffused Dextran-FITC in the lower chamber that passed through the endothelial barrier was measured by fluorescence intensity at 488 nm.

### mRNA isolation and real‑time PCR analysis of endothelial gap junction and tight junction protein expression

Total RNA was isolated by using the Monarch Total RNA Miniprep Kit (New England Biolabs, MA, USA) according to the manufacturer’s instructions. A total of 0.5 µg of the isolated total RNA was reverse transcribed via a High-Capacity cDNA Reverse Transcription Kit (Applied Biosystems, Waltham, MA, USA) according to the manufacturer’s instructions. Human *PECAM1*,* ZO1*,* CDH5* and *GAPDH* genes were analysed via real-time polymerase chain reaction (PCR) by using Fast-Start Essential DNA Green Master Mix (Roche, Basel, Switzerland) with specific primers (Table S2). *GADPH* was used as an internal control. The amount of target in the various samples was calculated via the 2^(−ΔCt) relative quantification method by using DataAssist v.3.01.

### Determination of the presence of tight junction proteins in HMEC-1 cells

HMEC-1 cells (4 × 10^5^) were seeded in the 24-well plates for 48 h. At 80–90% confluence, the full medium was changed by the medium without EGF and hydrocortisone for 16 h before the experiment began. The conditioned media of PMP-treated CRC cells (final concentration of 100 µg/mL) or PMP alone (final concentration of 100 µg/mL) were added to HMEC-1 cells for 24 h and the presence of PECAM-1 and ZO-1 proteins on the surfaces of endothelial cells was determined by using flow cytometry and confocal microscopy via rabbit polyclonal anti-human PECAM-1 antibody (Santa Cruz Biotechnology), rabbit monoclonal anti-human ZO-1 antibody (D7D12, Abcam) and secondary goat anti-rabbit antibody conjugated with Alexa Fluor488 (Invitrogen).

For the immunoblotting detection of total PECAM-1 and ZO-1 proteins, HMEC-1 cells were incubated for 24 h with PMP as described above and lysed in RIPA buffer (Sigma Aldrich). The lysates (20 µg of protein) were then separated on an SDS-PAGE 10% polyacrylamide gel, transferred to a nitrocellulose membrane and incubated with mouse anti-human antibodies against PECAM-1 (89C2; Cell Signaling, #3528) or rabbit anti-human antibodies against ZO-1 (Proteintech, 21773-1-AP), followed by incubation with secondary horse anti-mouse IgG (Cell Signaling, #7076) or goat anti-rabbit IgG (Invitrogen, #31460) conjugated with horseradish peroxidase (HRP).

### Transendothelial migration assay

HMEC-1 cells (4 × 10^5^) in full medium were seeded on 8 μm transwell inserts in a 24-well plate for 3 h. The migration of CRC cells (2 × 10^5^) labelled with CellTracker™ Green CMFDA dye through a monolayer of HMEC-1 cells that were previously preincubated with PMP (final concentration of 100 µg/ml) for 4 h in the experimental medium was determined after 24 h via fluorescence inverted microscope.

### Mouse model of colorectal cancer metastasis

All of the experiments were performed in accordance with the guidelines formulated by the European Community for the Use of Experimental Animals (L358-86/609/EEC) and the Guide for the Care and Use of Laboratory Animals published by the US National Institute of Health (NIH Publication No. 85–23, revised 1985). All of the procedures were approved by the Local Ethics Committee on Animal Experiments at the Medical University in Lodz (approval number: 38/Ł 241/2022). BALB/c nude mice (female, aged 6–8 weeks) were purchased from the sales distributor of Charles River Laboratories (Animalab). On the day of the experiment, the mice were anaesthetised via the intraperitoneal injection of a mixture of ketamine (100 mg/kg b/w/) and xylazine (10 mg/kg b.w.). The spleen was surgically exposed, and 15 µL of CRC cell suspension (1.5 × 10^6^ HT29, SW480 or SW620 cells in Hank’s balanced salt solution, 6 mice per CRC cell line) or HBSS alone (control, 4 mice) was injected into the middle part of the spleen with a Hamiltonian syringe. The mice were intravenously injected with PMP (20 µg protein in 100 µL PBS) or with PBS (control) every 7 days for the subsequent 5 weeks starting on the seventh day after intrasplenic injection. At the end of the experiment (on the 35th day), selected organs (including liver, spleen, colon and lungs) were surgically excised and macroscopically analysed by veterinarian to detect metastases.

### Histopathological examination

Mayer’s haematoxylin and eosin staining and the determination of CDX2, CK20 and CD41 were performed to microscopically detect metastases in paraffin-embedded samples of the liver, spleen, colon and lungs. Inflammatory changes in the liver were assessed using a semiquantitative 0–3 scale focused on inflammatory activity, which was based on the predominant portal or lobular involvement.

### Flow cytometric determination of platelet activation

Circulating platelet activation, and platelet reactivity in response to thrombin (final concentration of 0.25 U/mL) were evaluated based on the measured expressions of specific surface membrane antigens, including CD62P (P-selectin) and the activated αIIbβ3 complex, via specific rat anti-mouse antibodies, conjugated with PE (Emfret Analytics). Platelets were gated based on the binding of αIIbβ3 (nonactivated complex) antibodies conjugated with FITC.

### Quantification of cytokines in mouse plasma

Murine cytokines (IL-12p70, IL-10, IL-6, MCP-1, IFN-γ and TNF-𝛼) were detected in plasma via a commercial BD Cytometric Bead Array (CBA) Mouse Inflammation Kit, according to the manufacturer’s protocol.

### Enzyme-linked immunosorbent assay for MMP-2 and MMP-9

For the quantification of total MMP-2 and human MMP-9 levels in mice plasma, Total MMP-2 Quantikine ELISA Kit and Quantikine Human MMP-9 ELISA Kit were used (R&D Systems), according to the manufacturers’ protocols.

### Statistical analysis

The data are presented as the means and standard errors values. The normality of the data distributions was verified via the Shapiro-Wilk test, and variance homogeneity was tested via the Levene’s test. The Mann-Whitney U test was employed to evaluate the significance of the differences between two independent groups of variables not adhering to normality. Otherwise, the Student’s t test for independent samples was used to compare two groups. Statistical analysis was performed via GraphPad and StatsDirect.

## Results

### PMP can enhance CRC cell adhesion to endothelial cells, and this effect is abolished by the inhibition of clathrin- and caveolin-dependent endocytosis

In the bloodstream or in capillaries within the tumour microenvironment (TME), PMP can stimulate only ECs, only cancer cells or both types of cells simultaneously. Thus, for the analyses of CRC cell adhesion to the endothelium layer, we performed three sets of experiments with different initial steps; specifically, we used PMP to stimulate only CRC cells, only ECs, or both CRC cells and ECs simultaneously.

**Fig. 1:**
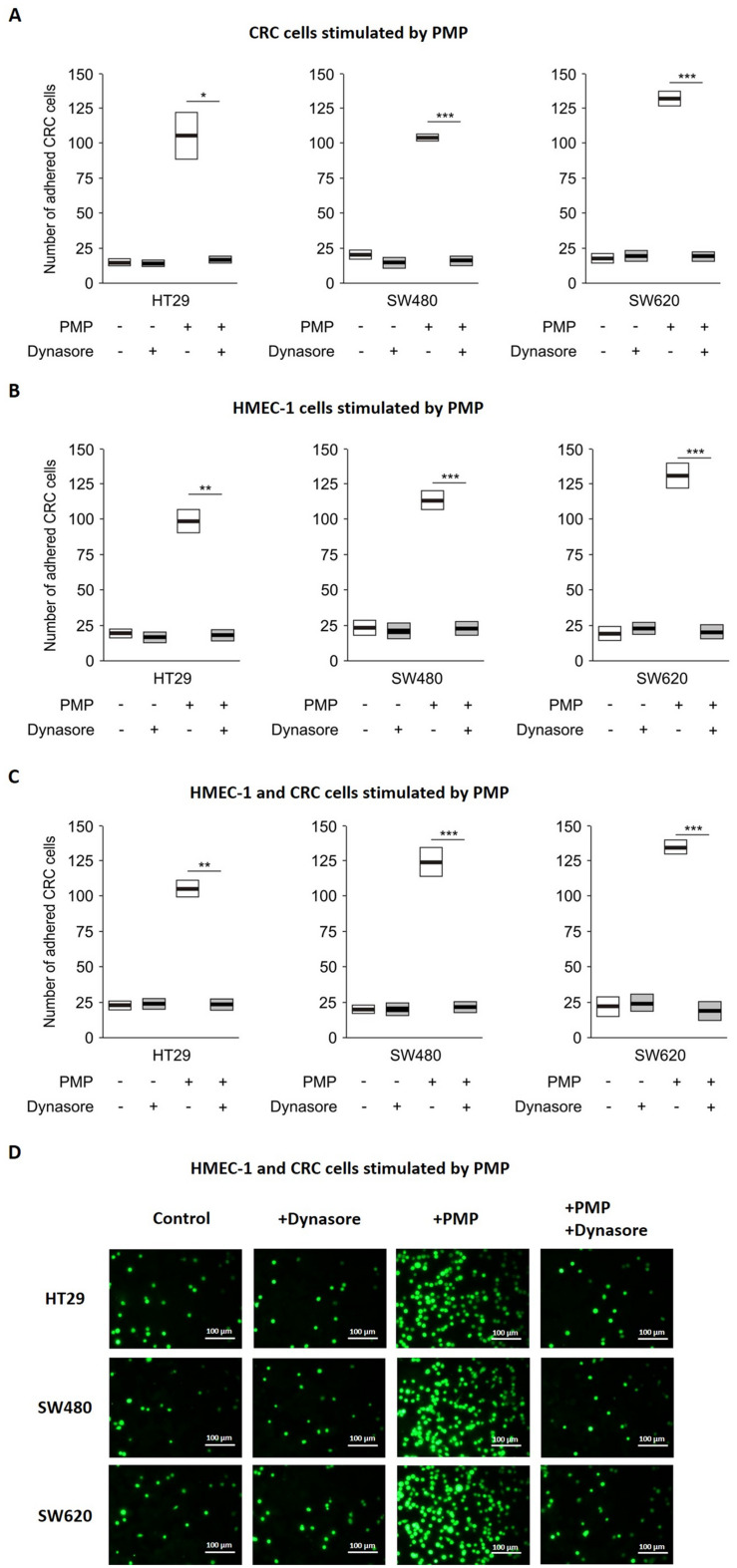
Inhibition of CRC cell adhesion to the HMEC-1 monolayer. **A, B, C **– Quantified data of the adhesion of CellTracker-labelled HT29, SW480 and SW620 cells to monolayers of HMEC-1 cells after 3 h of incubation. CRC cells alone (**A**), HMEC-1 cells alone (**B**) or both CRC cells and HMEC-1 cells (**C**) were preincubated with PMP (50 µg PMP/10^6^ cells at a final concentration of 100 µg/mL) and with or without the addition of the dynamine inhibitor, Dynasore (final concentration of 50 µM) or DMSO (final concentration of 0.1% as a control) for 4 h in appropriate medium not supplemented with FBS. The data are presented as the means (vertical lines) and standard errors (boxes) of the number of adhered, fluorescently labeled CRC cells. Statistics were calculated with a parametric Student’s t test: *** *P* < 0.001, ** *P* < 0.01, * *P* < 0.05, *N* = 3. **D **– Representative fluorescence microscopy images of CellTracker-labelled HT29, SW480 and SW620 CRC cells that adhered to monolayers of HMEC-1 cells after 3 h incubation. Both CRC cells and HMEC-1 cells were previously preincubated with PMP. Scale bars – 100 μm

The adhesion of CRC cells to the HMEC-1 monolayer under all three stimulation conditions was increased after PMP stimulation (Figs. 1 and S2). We also noted that the initial PMP preincubation step did not affect the adhesion level, which was comparable for each experimental set. These results indicated that the observed effect of PMP was not additive.

To investigate whether this effect depends on PMP endocytosis by CRC cells, we used the dynamin inhibitor; Dynasore (which is an inhibitor of clathrin-and caveolin endocytosis). First, we confirmed the inhibition of PMP endocytosis by Dynasore (Fig. S3). Subsequently, we used the same inhibitor to determine whether it affects CRC adhesion to the endothelial monolayer stimulated by PMP. As shown in Fig. 1, under all three experimental conditions, the inhibition of PMP endocytosis by Dynasore decreased the adhesion of CRC cells to HMEC-1 cells.

### PMP disrupt the integrity of endothelial cells and decrease the expression of intercellular junction proteins

Intravasation and extravasation depend not only on the adhesion and migration ability of cancer cells, but also on the disruption of vascular barrier integrity. An intact normal endothelium at distant sites can be preconditioned by cancer cells (due to released soluble factors or extracellular vesicles) and other factors (such as platelets or PMP).

**Fig. 2:**
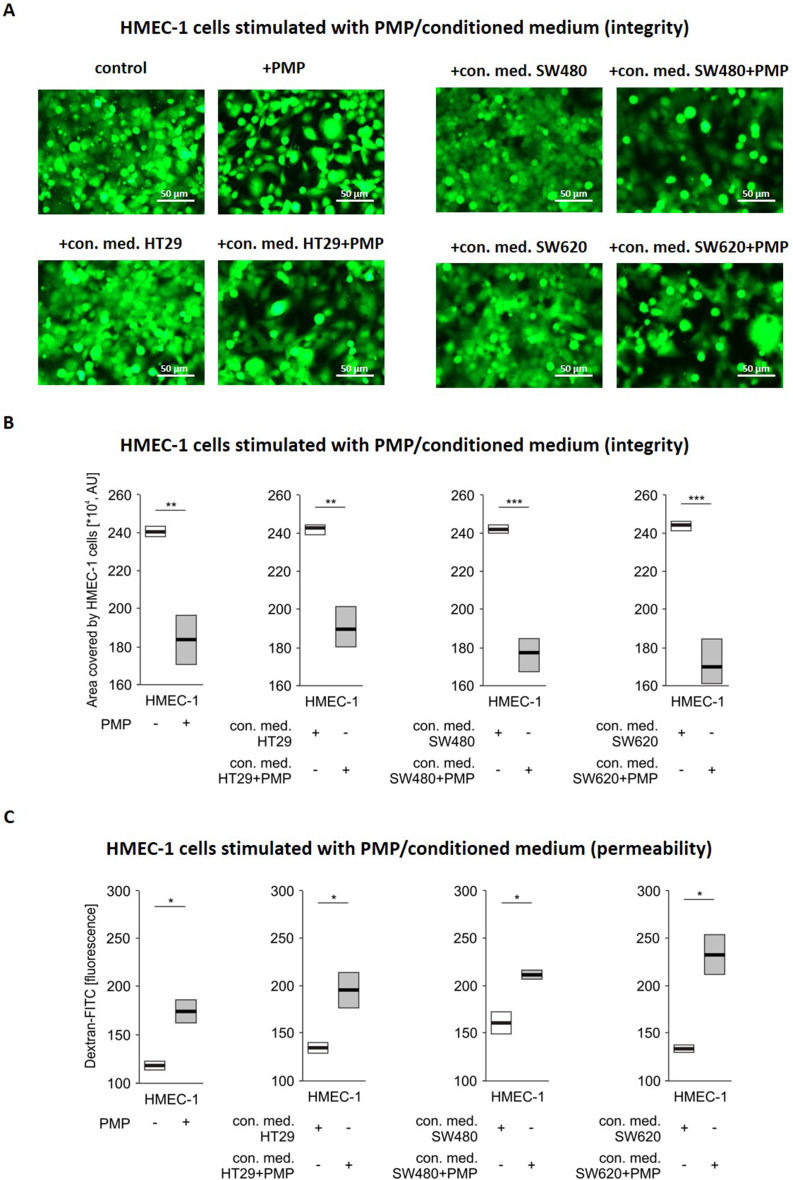
The effect of PMP and conditioned medium of PMP-stimulated CRC cells on the integrity of the HMEC-1 cell monolayer. **A **– Representative fluorescence microscopy images of integrity of CellTracker-labelled HMEC-1 cell monolayer. HMEC-1 cells were previously preincubated with PMP (final concentration of 100 µg/mL) or with conditioned medium (con. med.) (final protein conc. 100 µg/mL) from HT29, SW480 and SW620 cells that were either not stimulated (con. med. obtained from HT29, SW480, and SW620, respectively) or stimulated with PMP (con. med. obtained from HT29 + PMP, SW480 + PMP, and SW620 + PMP, respectively) for 4 h. Scale bars – 50 μm. **B **– Quantified data of HMEC-1 integrity are presented as the means (vertical lines) and standard errors (boxes) of the area covered with fluorescently labelled HMEC-1 cells. (**B**). Statistics were calculated by using a parametric Student’s t test or nonparametric Mann-Whitney test: ** *P* < 0.01,* *P* < 0.001, *N* = 3. **C **– Permeability of the HMEC-1 monolayer for the passage of Dextran-FITC. HMEC-1 cells were previously preincubated with PMP, as described above. The data are presented as the means (vertical lines) and standard errors (boxes) of fluorescence measured at 488 nm. Statistics were calculated by using parametric Student’s t test or nonparametric Mann-Whitney test: *** *P* < 0.05, *N* = 3

We investigated the direct influence of PMP on the integrity of the HMEC-1 monolayer and observed the disruption of monolayer integrity by PMP (Fig. 2). We also used the conditioned media of CRC cells after PMP stimulation to examine the indirect influence of PMP on endothelial monolayer integrity. As shown in Fig. 2, the incubation of ECs with CRC-conditioned media from all of the examined CRC cell lines led to disrupted continuity of the EC layer.

Due to the fact that the maintenance of vascular endothelial integrity is mediated by intercellular junctions [15], we subsequently examined whether PMP could modify the expression of the junction molecules of the endothelium.

**Fig. 3:**
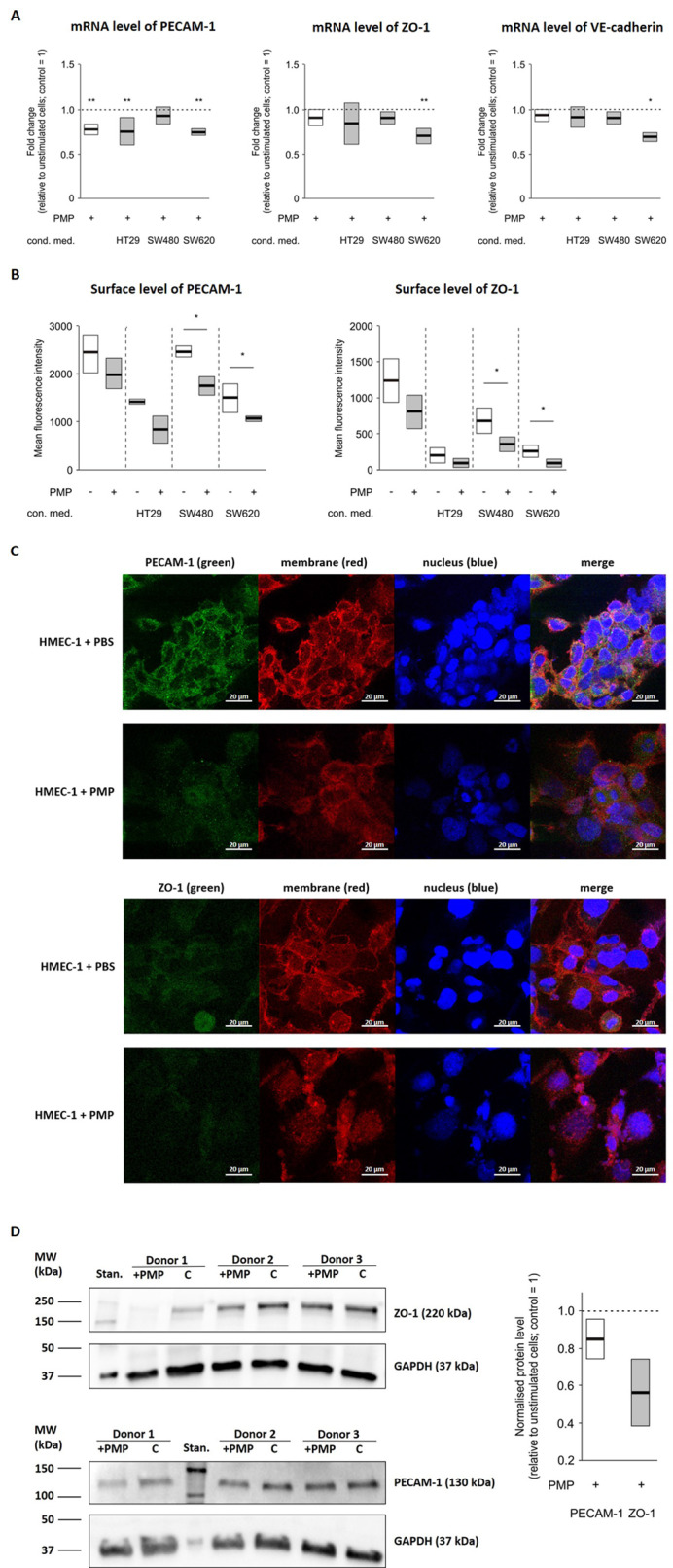
Expression of intercellular junction proteins in HMEC-1 cells treated with PMP or conditioned medium. **A **– mRNA expression of PECAM-1, ZO-1 and VE-cadherin in HMEC-1 cells treated with PMP or conditioned media from CRC cells previously treated with PMP. HMEC-1 cells were stimulated with PMP or conditioned media from appropriate controls. After 8 h, RNA was isolated and real-time PCR was performed. The results were calculated and are presented as the fold change in relative to that of unstimulated cells (control = 1, dashed line), *N* = 4–6. The data are presented as the means (vertical lines) and standard errors (boxes). Statistics were calculated by using a parametric Student’s t test or nonparametric Mann-Whitney test: **P < 0.01, *P < 0.05, *N* = 2–5. **B **– Flow cytometry analysis of PECAM-1 and ZO-1 on the surface of CRC cells incubated with PMP (final concentration of 100 µg/mL) or with conditioned media (final protein concentration of 100 µg/mL) from HT29, SW480 and SW620 cells that were either not stimulated or stimulated with PMP. The data are presented as the means (vertical lines) and standard errors (boxes). Statistics were calculated by using parametric Student’s t test or nonparametric Mann-Whitney test: *P < 0.05, *N* = 2–5. **C **– Representative confocal microscopy images of anti-PECAM-1 or anti-ZO-1 detection (green) on CRC cells treated with PMP for 24 h. Hoechst 33,342 (blue) was used to stain the nuclei and Alexa Fluor 594 (red) wheat germ agglutinin was used to stain the cell membrane. Scale bars – 20 μm. **D **– Representative Western blots of PECAM-1 (left upper panel) and ZO-1 (left lower panel) in HMEC-1 cells 24 h after PMP stimulation (left panel). Normalised densitometric values of PECAM-1 and ZO-1 in relative to those of unstimulated cells (control = 1, dashed line) (right panel); *N* = 3

Both, direct PMP treatment of HMEC-1 and treatment of HMEC-1 with conditioned media from PMP-treated CRC cells resulted in a transcriptional suppression of *PECAM1* (*p* = 0.002 for HMEC-1 + PBS vs. HMEC-1 + PMP; *p* = 0.001 for HMEC-1 + con. med. (HT29) vs. HMEC-1 + con. med. (HT29 + PMP); *p* = 0.002 for HMEC-1 + con. med. (SW620) vs. HMEC-1 + con. med. (SW620 + PMP)); *ZO1* (*p* = 0.002 for HMEC-1 + con. med. (SW620) vs. HMEC-1 + con. med. (SW620 + PMP)), and *CDH5* (*p* = 0.002 for HMEC-1 + con. med. (SW620) vs. HMEC-1 + con. med. (SW620 + PMP)) (Fig. 3A) in each CRC cell line. For further analysis, we performed flow cytometry to confirm whether PMP or conditioned media could modulate the level of surface protein expression. As shown in Fig. 3B, the protein levels of PECAM-1 and ZO-1 on the surface of HMEC-1 cells were significantly decreased after stimulation with conditioned media from SW480 (*p* = 0.03 for HMEC-1 + con. med. (SW480) vs. HMEC-1 + con. med. (SW480 + PMP); *p* = 0.04 for HMEC-1 + con. med. (SW620) vs. HMEC-1 + con. med. (SW620 + PMP), respectively) and SW620 cells (*p* = 0.03 for HMEC-1 + con. med. (SW480) vs. HMEC-1 + con. med. (SW480 + PMP); *p* = 0.04 for HMEC-1 + con. med. (SW620) vs. HMEC-1 + con. med. (SW620 + PMP), respectively). We also observed the same trend after incubation of HMEC-1 cells with PMP and conditioned medium from HT29 cells. We confirmed the reduction in the surface levels of the PECAM-1 and ZO-1 proteins via confocal microscopy (Fig. 3C). The tendency towards reduced total ZO-1 and PECAM-1 protein levels in HMEC-1 cells stimulated with PMP was revealed via Western blot (Fig. 3D).

### PMP promote the transendothelial migration of CRC cells

**Fig. 4:**
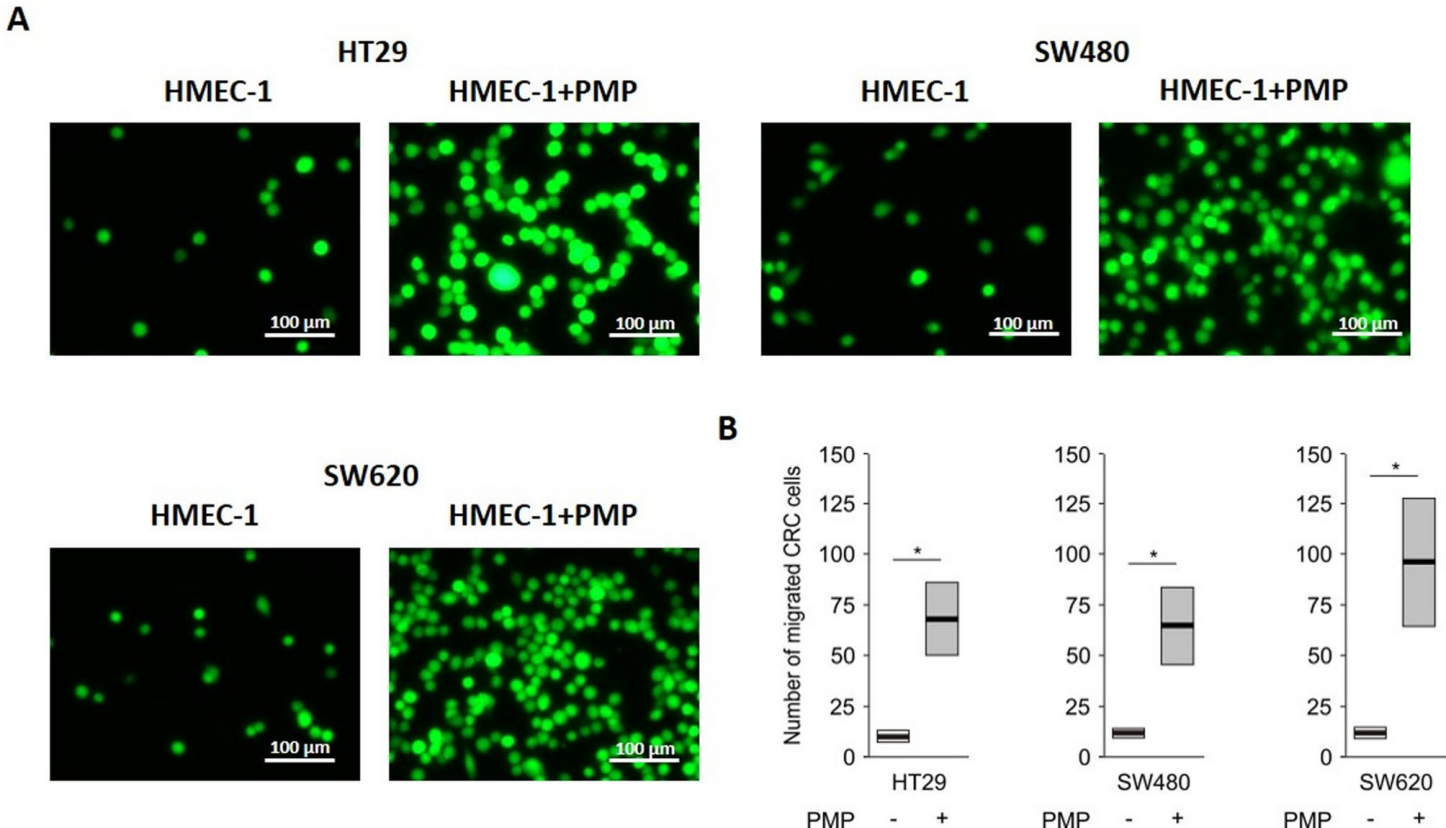
The effects of PMP on the migration of CRC cells through the HMEC-1 monolayer. **A **– Representative fluorescence microscopy images of CellTracker-labelled HT29, SW480 and SW620 cells that migrated through monolayers of HMEC-1 cells in transwell chambers after 24 h of incubation. HMEC-1 cells were previously preincubated with PMP (final concentration 100 µg/mL) for 4 h. Scale bars – 100 μm. **B **– Transendothelial CRC cell migration is presented as the means (vertical lines) and standard errors (boxes) of the number of migrated cells. Statistics were calculated by using a parametric Student’s t test: * *P* < 0.05, *N* = 3

Due to the fact that the disruption of vascular barrier integrity is crucial to cell transmigration, we subsequently examined the effect of PMP on the transendothelial migration of CRC cells. Figure 4 shows that incubation of HMEC-1 cells with PMP increased the migration of fluorescently labelled CRC cells through HMEC-1 cells. Moreover, the number of all tested migrated CRC cells was increased, which demonstrates that PMP can enhance transendothelial migration regardless of the cancer cell phenotype.

### Colorectal cancer metastases are observed after intrasplenic injections of cells in a mouse model of liver metastasis

For in vivo experiments, we utilised intrasplenic injections of two human CRC cell lines known as SW480 and SW620, as they are derived from different stages of colon carcinoma in the same patient, thus representing interesting models for studies focusing on the late stages of CRC progression [16, 17]. The intrasplenic injections were followed by intravenous injections of PMP or PBS as a control. In our study, we used pooled PMP suspensions isolated from five different human platelet concentrates. Moreover, we used nude mice to prevent xenograft rejection in an implantation model of CRC.

**Table 1:** Incidences of liver metastases and other pathological abnormalities in mice that received intrasplenic injections of SW480 or SW620 cells followed by injections of PMP. For abnormalities other than liver abnormalities, see table S1

Group	Cell injection on day 0	PMP/PBS injections every 7 days	Number of liver metastases	Inflammation in the liver
1	HBSS	PBS	0/4 (0%)	0/4
2	SW480	PBS	4/5 (80%)	1/5 (20%)
3	SW480	PMP	5/5 (100%)	1/5 (20%)
4	SW620	PBS	2/6 (33%)	0/6 (0%)
5	SW620	PMP	4/6 (67%)	2/6 (33%)

HBSS: Hank’s balanced salt solution

**Fig. 5:**
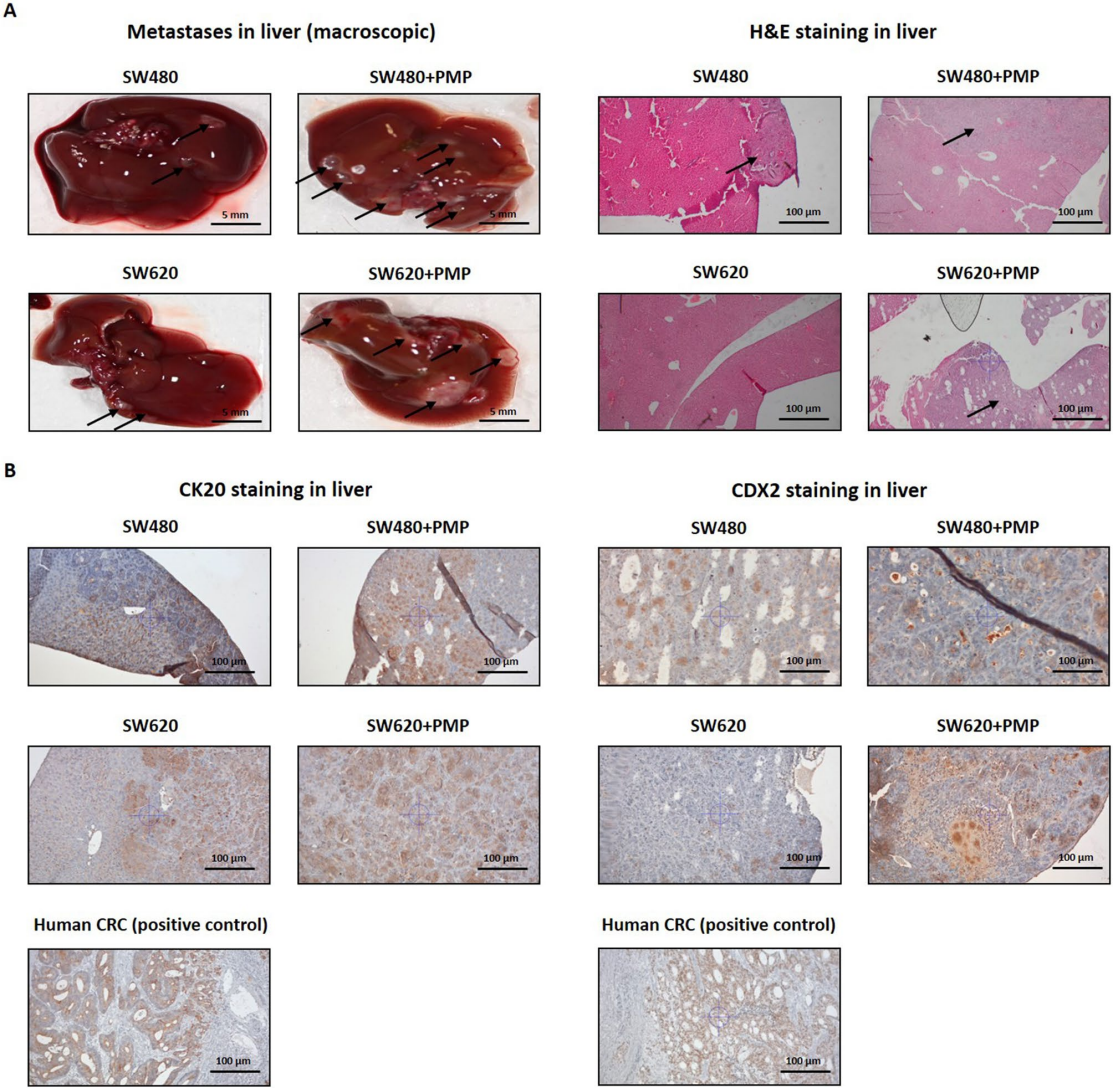
The effects of PMP on liver metastasis in an in vivo CRC model. **A**, left panel – Representative macroscopic images of livers obtained from mice 6 weeks after the administration of HBSS (control), SW480 cells or SW620 cells (1.5 × 10^6^ CRC cells in 15 µL of HBSS/mouse) and the intravenous administration of PBS (control) or PMP (20 µg in 100 µL of PBS/mouse). The black arrows indicate metastases. Scale bars – 5 mm. A, right panel – Representative microscopy images of H&E-stained samples of mouse livers from mice treated with selected lines of CRC cells and intravenously injected with PMP. The black arrows indicate metastases. Scale bars – 100 μm. **B **– Representative microscopy images of CK20 (left panel)-stained samples and CDX2 (right panel)-stained samples of livers obtained from a CRC mouse model (induced by the intrasplenic administration of SW480 or SW620 cells) intravenously injected with PBS (control) or PMP. Human CRC samples served as positive controls. Scale bars – 100 μm

Liver metastasis formation was macroscopically confirmed in mice injected with the tested CRC cell lines and via histochemical (H&E) and immunohistochemical (CK20 and CDX2) analyses (Table 1; Fig. 5A and B). However, we observed various degrees of metastasis formation, including 90% metastasis of SW480 cells (% mean from Groups 2 and 3, Table 1) and 50% metastasis of SW620 cells (% mean from Groups 4 and 5, Table 1). The administration of PMP differentially affected the number of liver metastases in a cell line-dependent (Table 1), with a more significant prometastatic effect of PMP being observed in the group of mice inoculated with SW620 cells and 67% metastatic effect being observed in the group of mice treated with PMP, compared to 33% metastatic effect observed in the group of mice treated with PBS (Groups 4 and 5, Table 1). In all of the groups, we also detected abnormalities such as enlarged lymph nodes, peritoneal metastases or thickening of the intestinal walls (Table S1).

### PMP injections induce prometastatic changes during cancer progression in vivo

**Fig. 6:**
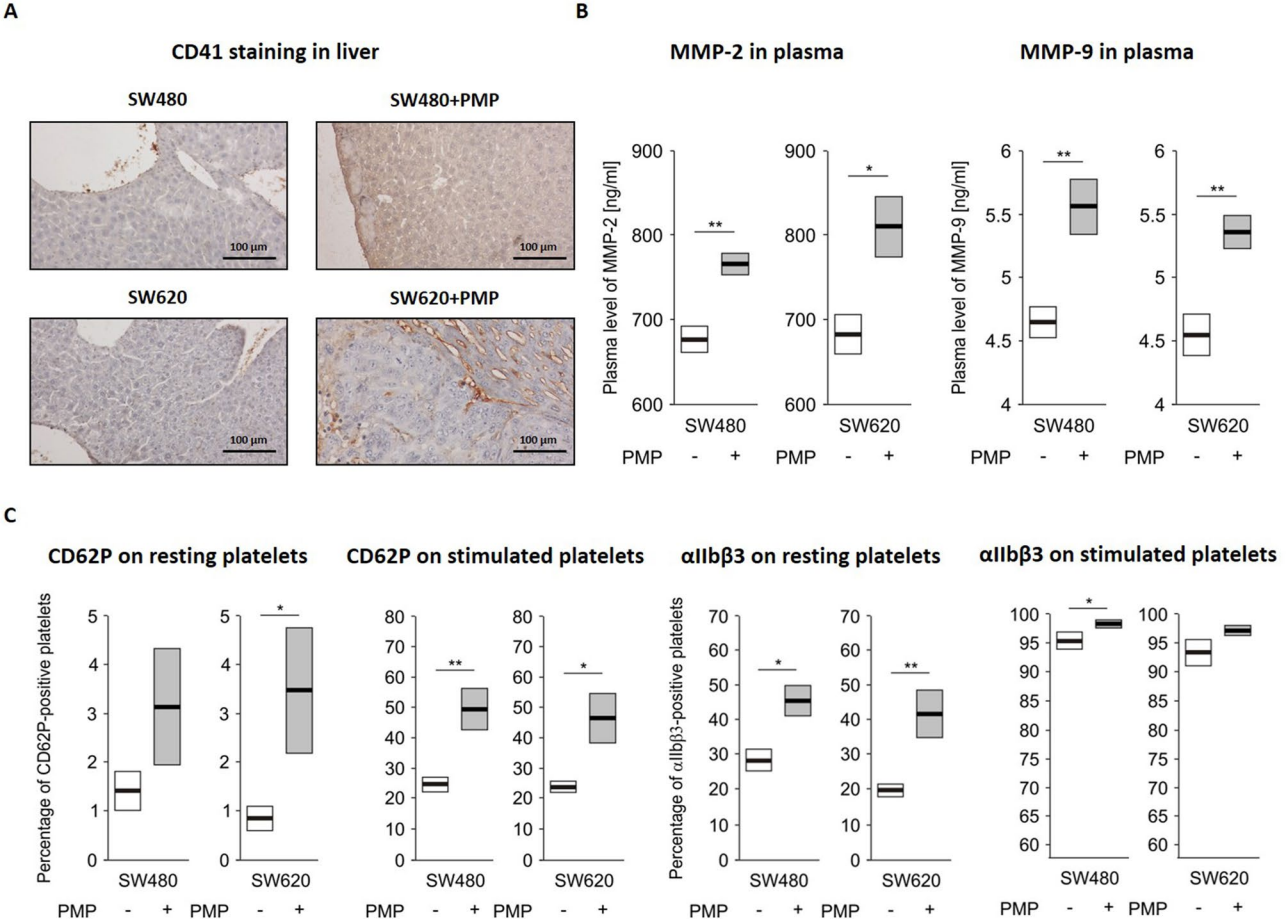
The effects of PMP on cancer cell invasiveness and platelet activation in an in vivo CRC model. **A **– Representative microscopy images of anti-human CD41-stained samples of mouse livers from mice treated with selected lines of CRC cells and intravenously injected with PMP. Scale bars – 100 μm. **B **– Concentrations of total MMP-2 and human MMP-9 in the plasma obtained from mice treated with selected lines of CRC cells and intravenously injected with PMP. **C **– Expression of CD62P (presented as a percentage of CD62P-positive platelets) and αIIbβ3 (presented as a percentage of αIIbβ3-positive platelets), in resting and thrombin-stimulated platelets obtained from a CRC mouse model intravenously injected with PMP. The quantified data are presented as the means (vertical lines) and standard errors (boxes). Statistics were calculated by using a parametric Student’s t test or non-parametric Mann-Whitney test: ** *P* < 0.01, * *P* < 0.05, *N* = 5–6

First, we analysed whether PMP are present in the formed metastases and identified their localisation in cancer tissues. Immunohistochemical analysis of the metastatic lesions in the liver via an antibody against the human platelet glycoprotein llb/CD41, which does not cross-react with mouse CD41, revealed that PMP are localized on the endothelial-mesenchymal margin or within the tumour endothelium (Fig. 6A).

In our previous study, we reported that PMP can increased the expression and activity of MMP-2 and MMP-9, thereby leading to the stimulation of CRC cell invasive activity in vitro. To verify whether PMP induce metastasis in vivo by influencing MMPs, we also investigated the impacts of PMP on the levels of human MMP-9 and total MMP-2 in mouse plasma in terminally collected blood from experimental mice (day 35 ). In mouse plasma, we detected elevated levels of humanMMP-9 and total MMP-2 after mice injections with PMP (Fig. 6B).

The inflammatory processes that occur during CRC development are undesirable because they accelerate tumour growth in many cases. Thus, we also analysed the plasma of the animals collected at the end of the experiment, for the presence of the inflammatory cytokines IL-12p70, IL-10, IL-6, MCP-1, IFN-γ and TNF-𝛼. We did not observe any changes in the plasma concentrations of the investigated cytokines in the animals treated with PMP (Fig. S4).

In addition to measuring inflammatory cytokines in mouse plasma, we also performed histopathological examinations of paraffin-embedded mouse livers to identify changes in tissue structures that are characteristic of inflammation, based on the predominant portal or lobular involvement. The entire procedure was performed by a professional histopathologist. Despite the lack of observations of the effect of PMP on systemic inflammation, we detected the inflammatory changes in the livers of the mice, which were dependent on the specific CRC line (Fig. 5 and Fig. S5, left). In our study, we did not identify any metastatic sites in the lungs of the mice at 5 weeks after the initial injection, and the administration of PMP did not affect the outcome. However, early interstitial pneumonia was observed in animals injected with both CRC lines, with at least 67% of the mice being affected in each group (Fig. S5 right).

### Platelet activation in a mouse model of liver metastasis is increased by PMP stimulation

To investigate the effect of the presence of PMP in the circulation, we evaluated platelet activation (activation of circulating resting platelets) and reactivity (after ex vivo activation in response to an agonist, thrombin) in terminally collected blood from experimental mice (day 35) via measurements of selectin P (CD62P) and integrin αIIbβ3 (activated complex) levels on platelets. CD62P and activated integrin αIIbβ3 are commonly used as markers for determining platelet activation in physiological states [18] and in cancer [19]. We observed that injections of human PMP increased the surface expression of both markers and the sensitivity of platelets to agonist stimulation (Fig. 6C). We also observed a statistically significant increase in the surface level of selectin P in thrombin-activated (but not resting) platelets (Fig. 6C, left panel) in all of the groups of mice treated with PMP. Moreover, we also observed an increasing trend in surface αIIbβ3 integrin (activated complex) levels in both resting and thrombin-activated platelets (Fig. 6C, right panel).

## Discussion

Extracellular vesicles (EVs) have attracted interest because they play a role in numerous diseases, including infectious, inflammatory and cardiovascular diseases, as well as cancer progression [20–22]. Approximately 70–90% of all EVs in the peripheral blood accounts for PMP [23, 24]. Among other EVs, the signature of PMP depends on the CRC progression and this signature is different before and after CRC remission [25]. A positive correlation between PMP and the presence of lymph node metastases in CRC patients has also been shown [26].

Our group recently reported that PMP can be incorporated by CRC cells of different phenotypes (including epithelial-like and mesenchymal-like phenotypes) and it promotes the invasion abilities of CRC cell lines by influencing the expression and activity of the metalloproteases MMP-2 and MMP-9 [7]. In this study, we focused on investigating the influence of PMP on the transendothelial migration (transmigration) of CRC cells. Transmigration is indispensable in metastatic events [27]. Intravasation and extravasation involve the specific interaction of cancer cells with vascular ECs via initial attachment, followed by stable adhesion, thus resulting in the transmigration of cancer cells [28]. By utilising clathrin- and caveolin-dependent endocytosis inhibitors, we demonstrated that PMP uptake by either CRC cells, ECs or both cell types is necessary to increase the adhesion of CRC cells to ECs.

We assume that in the circulation PMP influence cancer cells and ECs in at least two steps. First, due to the increased number of platelets in the peripheral blood (such as blood obtained from healthy donors due to transfusions after postsurgical treatment) can be activated by CRC cells, thereby leading to the release of PMP. Second, after incorporation by circulating CRC cells, the increased number of PMP stimulates the cells to release various factors, which can subsequently affect the integrity of the endothelial cell layer. These situations mirror the reciprocal and complex interactions occurring among CRC cells, ECs and PMP released by PLT. Therefore, we focused on the endothelial integrity in two scenarios: EC stimulation by PMP or stimulation with conditioned medium from CRC cells that were previously stimulated with PMP. Under both conditions we confirmed that PMP could decrease EC integrity.

Given that adhesive intercellular contacts are crucial for the regulation of microvascular function, which includes vascular permeability and the transendothelial migration of cancer cells [29], we confirmed that PMP could modify the levels of adhesion proteins VE‑cadherin, PECAM-1 and ZO-1 in ECs. We detected decreases in the transcript and cell surface levels of these proteins,. VE‑cadherin is the main transmembrane component of adherent junctions in ECs [30]. Moreover, the destabilization of the membrane distribution of VE-cadherin results in increased permeability of ECs [31]. One mechanism responsible for the dissociation of the VE-cadherin/beta-catenin complex cells is the activation of p38 and ERK in endothelial cells treated with cancer, which initiate the activation of Src kinase activities [32].

Another protein located on the surfaces of EC cells that is inhibited by the presence of PMP and conditioned media is platelet-endothelial adhesion molecule-1 (PECAM-1; CD31). The expression of this protein is largely concentrated at junctions between adjacent and endothelial cells [3]. PECAM-1 has important functions in the endothelium, such as cell-cell adhesion, cell signaling, and barrier function [33]. A study of the role of retinal microvascular PECAM-1 in a type 1 diabetic rat model, as well as in cultured retinal ECs in hyperglycaemic media, reported a significant decrease in PECAM-1 protein levels, which was dependent on MMPs [34]. Our previous observation [7] that PMP (when incorporated into CRC cells) stimulate the metalloproteases MMP-2 and MMP-9 (which are able to degrade the extracellular matrix) may be consistent with the findings of studies focusing on hyperglycaemia. Moreover, a previous study of non-small cell lung cancer patients revealed that high (rather than low) expression of PECAM-1 was associated with improved survival [35].

The aim of our study was to determine the conditions that are most similar to those that are predominant in living organisms, thus reflecting the situation in which PMP can stimulate CRC cells to release different factors after they are incorporated into CRC cells. Thus, we are aware that the conditioned media prepared in this study may contain PMP. However, in our previous study revealed that the uptake of PMP by CRC cells increased over time [7]. Therefore, we believe that the number of PMP present in the conditioned media is strongly reduced and that the effect of the conditioned media on the integrity of the endothelial cell layer is due to soluble factors (such as MMPs), as shown in [7].

Similarly, decreased levels of the tight junction protein ZO-1 have been reported in studies of EC permeability [36], and EVs can exert an influence on EC integrity [37] during breast cancer progression. Taken together, our in vitro results strongly suggest that PMP reduce the levels of adhesion proteins that are involved in the maintenance of endothelial integrity, thus providing a direction for future research on how to avoid the negative role of PMP in cancer progression.

The results obtained from the in vitro experiments prompted us to evaluate the ability of PMP to modulate CRC cells abilities that lead to metastasis in a mouse model in vivo. In the mouse model, CRC cells were not directly injected into the circulation; instead they had to undergo all steps of metastasis, including transendothelial migration (transmigration). The main goal in the treatment of CRC is to prevent the formation of metastases. The mortality of CRC patients is particularly associated with the occurrence of metastases in the liver or lungs [38]. We used PMP obtained by the activation of platelets from healthy donors’ platelet-rich plasma concentrates, as PMP can be formed in the patient’s body both via the activation of circulating platelets and via delivery during blood or platelet transfusion in postsurgery CRC therapies. The dose of PMP that was administered to the animals in this study was based on other reports regarding in vivo injections of cellular microvesicles [39]. Moreover, we employed a model of intrasplenic injection of human CRC cells into immunodeficient mice. The intrasplenic model is a well-known and commonly accepted animal model of colorectal cancer cell metastasis to the liver [40]. As expected, we observed the metastasis of CRC cells, which primarily occurred to the liver. We did not observe any metastatic sites in the lungs; however, we detected metastatic lesions in the peritoneum, which are also common in patients with CRC [41]. In our study, the administration of PMP affected the number of animals with liver metastases, and we observed an increase in the number of mice bearing SW620 tumours. This finding is not surprising due to the fact that this cell line is already derived from a lymph node metastasis patient, whereas SW480 represents primary colon adenocarcinoma [42]. At this point, we suggest a supporting (rather than triggering) role of PMP in CRC progression.

We observed the presence of human PMP on the margins of the metastatic sides and tumour capillaries. The same observations have been reported in images from human colon cancer array with paired uninvolved tissue where, due to dysfunctional EC and small diameter of endothelial capillaries, PMP are present in the tissue adjacent to the tumour border [6]. We hypothesised that PMP released from PLT can modulate CRC cell-endothelial cell interactions during intravasation and extravasation events, which leads to the metastatic spread of cells within organisms. Our results align with the findings of an earlier report demonstrating that the interaction of PLT with CRC cells induces EMT and increases the ability of tumour cells to adhere to the endothelium [43].

In addition to the outcome of the formation of metastases in the liver, we also analysed the blood collected at the end of the experiment to determine the influence of PMP on plasma metalloprotease concentrations. We observed an increase in human MMP-2 and total MMP-9 concentrations in mouse plasma after injections with PMP. Platelets contain MMP-2 and MMP-9, as has been demonstrated in the literature [44, 45]; however, there are no reports demonstrating that pro-MMP-2, pro-MMP-9, MMP-2 and MMP-9 are the cargo molecules of PMP. The levels of these proteins are increased after the treatment of breast, lung and prostate cancer cells with PMP [46–48], and this effect is explained by the upregulation of MMP production by PMP. As we observed in the previous study [7], MMP-9 is increased in CRC cell lines after stimulation with PMP, but is not present in PMP alone. Therefore, we can conclude that elevated levels of human MMP-2 and MMP-9 in mouse plasma result from increased release of these metalloproteases from PMP-stimulated CRC cells in vivo.

Proinflammatory factors trigger the formation of an early premetastatic niche that is characterised by ECM remodelling and inflammatory changes which may lead to development of organ-specific metastases [49]. Thus, we analysed the impacts of PMP on systemic inflammation and liver and lung inflammation statutes in mice (as these organs serve as the most common sites of metastatic lesions observed in CRC, along with the peritoneum). We did not observe any effect of PMP on systemic inflammation; however, liver inflammation upregulation was observed after PMP injections in the groups of mice with tumours from more metastatic cell lines. Early interstitial pneumonia was also observed; however, this effect was independent of PMP.

The activation of circulating resting platelets and their reactivity after ex vivo activation in response to agonist, were assessed by measuring selectin P and integrin αIIbβ3 (activated complex) levels on mouse platelets. Selectin P and integrin αIIbβ3 are platelet surface molecules that mediate interactions between platelets and ECs as well as PLT rolling and arrest, respectively [50]. When considering the species differences between the administered human PMP/CRC cells and circulating mouse platelets, it is unlikely that the observed changes in platelet activation resulted from a direct interaction between PMP/CRC cells and mouse PLT. More likely human cancer cells that form observed metastases, are affected by administrated human PMP and consequently release factors that can trigger platelet activation [10]. Another possible mechanism of PLT activation (which includes PMP release) is its influence on the endothelium [51–53].

Some critical limitations of the in vivo model used in our study should be emphasised. One limitation is that we intravenously administered PMP. PMP are present in the circulation for only a short period of time and can be taken up not only by CRC cells, but also by other cells present in the bloodstream. Our methodology is supported by other reports, in which different types of extracellular vesicles have been intravenously administered [54–59]. To improve the presence of PMP in the circulation, we applied a series of PMP injections per mouse throughout the experimental period. We believe that the use of such approach (rather than the use of a single administration of PMP) would provide conditions in which more exogenic PMP are present in the circulation.

## Conclusions

In conclusion, we demonstrated that PMP promote CRC cell adhesion to ECs, which contributes to cancer cell extravasation. PMP also disrupt the integrity of endothelial cell junctions and enhanced the transendothelial migration of CRC cells. Thus, we deduce that PMP can modulate CRC cell-endothelial cell interactions during intravasation and extravasation events, which lead to the metastatic spread of cells within organisms. Moreover, our in vivo study revealed that PMP promote metastatic events by increasing the levels of circulating metalloproteases and increasing liver metastasis in mice.

## Electronic supplementary material

Below is the link to the electronic supplementary material.

Supplementary Material 1

## Data Availability

The datasets that were used and/or analysed during the current study are available from the corresponding author upon reasonable request.

## Declarations

### Tumour size

The maximal tumour size in mice permitted by the ethics committee in Poland is 12 mm. This scenario applies only to animal models in which cancer cells are subcutaneously injected. In the case of tumour induction via the injection of cancer cells into the organs of mouse (intrabody injections ) there are no specific guidelines for tumour size provided by the ethics committee in Poland. In the present study, we performed intrasplenic (intraorgan ) injections of cancer cells into immunodeficient mice. Regardless, in our study the maximum tumour size did not exceed the guidelines for subcutaneously induced tumours.

## Abbreviations

CRC: Colorectal cancer
EMT: Epithelial-to-mesenchymal transition
EVs: Extracellular vesicles
HBSS: Hank’s balanced salt solution
MMPs: Metalloproteases
PCR: Polymerase chain reaction
PLT: Platelets
PMP: Platelet-derived microparticles
TME: Tumour microenvironment

## References

[1] American Society of Clinical Oncology. Colorectal Cancer: Statistics Cancer.Net 2005–2020 American Society of Clinical Oncology (ASCO); 2020 [updated 01/2020. Available from: https://www.cancer.net/cancer-types/colorectal-cancer/statistics

[2] Paschos KA, Majeed AW, Bird NC. Natural history of hepatic metastases from colorectal cancer–pathobiological pathways with clinical significance. World J Gastroenterol. 2014;20(14):3719–37. 24744570 10.3748/wjg.v20.i14.3719 PMC3983432

[3] Carlos TM, Harlan JM. Leukocyte-endothelial adhesion molecules. Blood. 1994;84(7):2068–101. 7522621

[4] Choi H, Moon A. Crosstalk between cancer cells and endothelial cells: implications for tumor progression and intervention. Arch Pharm Res. 2018;41(7):711–24. 29961196 10.1007/s12272-018-1051-1

[5] Lazar S, Goldfinger LE. Platelets and extracellular vesicles and their cross talk with cancer. Blood. 2021;137(23):3192–200. 33940593 10.1182/blood.2019004119 PMC8351904

[6] Michael JV, Wurtzel JGT, Mao GF, Rao AK, Kolpakov MA, Sabri A, et al. Platelet microparticles infiltrating solid tumors transfer MiRNAs that suppress tumor growth. Blood. 2017;130(5):567–80. 28500171 10.1182/blood-2016-11-751099 PMC5542851

[7] Kassassir H, Papiewska-Pająk I, Kryczka J, Boncela J, Kowalska MA. Platelet-derived microparticles stimulate the invasiveness of colorectal cancer cells via the p38MAPK-MMP-2/MMP-9 axis. Cell Commun Signal. 2023;21(1):51. 36882818 10.1186/s12964-023-01066-8 PMC9990213

[8] Li X, Ma Y, Liu C, Pu F, Zhang Y, Wang D. Platelet membrane-derived microparticles May be biomarkers in patients with hepatocellular carcinoma and can promote the invasion and metastasis of hepatoma carcinoma cells. Transfusion. 2023;63(10):1821–31. 37680187 10.1111/trf.17499

[9] Vismara M, Zarà M, Negri S, Canino J, Canobbio I, Barbieri SS, et al. Platelet-derived extracellular vesicles regulate cell cycle progression and cell migration in breast cancer cells. Biochim Biophys Acta Mol Cell Res. 2021;1868(1):118886. 33039555 10.1016/j.bbamcr.2020.118886

[10] Zarà M, Guidetti GF, Boselli D, Villa C, Canobbio I, Seppi C, et al. Release of prometastatic Platelet-Derived microparticles induced by breast Cancer cells: A novel positive feedback mechanism for metastasis. TH Open. 2017;1(2):e155–63. 31249921 10.1055/s-0037-1613674 PMC6524851

[11] John A, Tuszynski G. The role of matrix metalloproteinases in tumor angiogenesis and tumor metastasis. Pathol Oncol Res. 2001;7(1):14–23. 11349215 10.1007/BF03032599

[12] Wang Y, Wei Y, Huang J, Li X, You D, Wang L, et al. Prognostic value of matrix metalloproteinase-2 protein and matrix metalloproteinase-9 protein in colorectal cancer: a meta-analysis. BMC Cancer. 2024;24(1):1065. 39210344 10.1186/s12885-024-12775-9 PMC11360742

[13] Kryczka J, Kassassir H, Papiewska-Pająk I, Boncela J. Gelatin in situ zymography to study gelatinase activity in Colon cancer cells treated with platelet microparticles (PMPs). Methods Mol Biol. 2024;2747:167–76. 38038940 10.1007/978-1-0716-3589-6_14

[14] Martins-Green M, Petreaca M, Yao M. An assay system for in vitro detection of permeability in human endothelium. Methods Enzymol. 2008;443:137–53. 18772015 10.1016/S0076-6879(08)02008-9

[15] Komarova YA, Kruse K, Mehta D, Malik AB. Protein interactions at endothelial junctions and signaling mechanisms regulating endothelial permeability. Circ Res. 2017;120(1):179–206. 28057793 10.1161/CIRCRESAHA.116.306534 PMC5225667

[16] Hewitt RE, McMarlin A, Kleiner D, Wersto R, Martin P, Tsokos M, et al. Validation of a model of colon cancer progression. J Pathol. 2000;192(4):446–54. 11113861 10.1002/1096-9896(2000)9999:9999<::AID-PATH775>3.0.CO;2-K

[17] Nakurte I, Jekabsons K, Rembergs R, Zandberga E, Abols A, Linē A, et al. Colorectal Cancer cell line SW480 and SW620 released extravascular vesicles: focus on Hypoxia-induced surface proteome changes. Anticancer Res. 2018;38(11):6133–8. 30396929 10.21873/anticanres.12965

[18] Schmidt GJ, Reumiller CM, Ercan H, Resch U, Butt E, Heber S, et al. Comparative proteomics reveals unexpected quantitative phosphorylation differences linked to platelet activation state. Sci Rep. 2019;9(1):19009. 31831789 10.1038/s41598-019-55391-5 PMC6908631

[19] Xue J, Deng J, Qin H, Yan S, Zhao Z, Qin L, et al. The interaction of platelet-related factors with tumor cells promotes tumor metastasis. J Transl Med. 2024;22(1):371. 38637802 10.1186/s12967-024-05126-6 PMC11025228

[20] Cui Z, Zhang L, Hu G, Zhang F. Extracellular vesicles in cardiovascular pathophysiology: communications, biomarkers, and therapeutic potential. Cardiovasc Toxicol. 2024. 10.1007/s12012-024-09875-0 38844744

[21] Rahmati S, Moeinafshar A, Rezaei N. The multifaceted role of extracellular vesicles (EVs) in colorectal cancer: metastasis, immune suppression, therapy resistance, and autophagy crosstalk. J Transl Med. 2024;22(1):452. 38741166 10.1186/s12967-024-05267-8 PMC11092134

[22] Zhang L, Chi J, Wu H, Xia X, Xu C, Hao H, et al. Extracellular vesicles and endothelial dysfunction in infectious diseases. J Extracell Biol. 2024;3(4):e148. 38938849 10.1002/jex2.148 PMC11080793

[23] Berckmans RJ, Nieuwland R, Böing AN, Romijn FP, Hack CE, Sturk A. Cell-derived microparticles circulate in healthy humans and support low grade thrombin generation. Thromb Haemost. 2001;85(4):639–46. 11341498

[24] Italiano JE Jr., Mairuhu AT, Flaumenhaft R. Clinical relevance of microparticles from platelets and megakaryocytes. Curr Opin Hematol. 2010;17(6):578–84. 20739880 10.1097/MOH.0b013e32833e77ee PMC3082287

[25] Mege D, Panicot-Dubois L, Ouaissi M, Robert S, Sielezneff I, Sastre B, et al. The origin and concentration of Circulating microparticles differ according to cancer type and evolution: A prospective single-center study. Int J Cancer. 2016;138(4):939–48. 26341361 10.1002/ijc.29837

[26] Dymicka-Piekarska V, Gryko M, Lipska A, Korniluk A, Siergiejko E, Kemona H. Platelet-Derived microparticles in patients with colorectal Cancer. J Cancer Therapy. 2012;03(06):898–901.

[27] Chiang SP, Cabrera RM, Segall JE. Tumor cell intravasation. Am J Physiol Cell Physiol. 2016;311(1):C1–14. 27076614 10.1152/ajpcell.00238.2015 PMC4967137

[28] Di Russo S, Liberati FR, Riva A, Di Fonzo F, Macone A, Giardina G, et al. Beyond the barrier: the immune-inspired pathways of tumor extravasation. Cell Commun Signal. 2024;22(1):104. 38331871 10.1186/s12964-023-01429-1 PMC10851599

[29] Reymond N, d’Água BB, Ridley AJ. Crossing the endothelial barrier during metastasis. Nat Rev Cancer. 2013;13(12):858–70. 24263189 10.1038/nrc3628

[30] Breviario F, Caveda L, Corada M, Martin-Padura I, Navarro P, Golay J, et al. Functional properties of human vascular endothelial Cadherin (7B4/cadherin-5), an endothelium-specific Cadherin. Arterioscler Thromb Vasc Biol. 1995;15(8):1229–39. 7627717 10.1161/01.atv.15.8.1229

[31] Muramatsu F, Kidoya H, Naito H, Hayashi Y, Iba T, Takakura N. Plakoglobin maintains the integrity of vascular endothelial cell junctions and regulates VEGF-induced phosphorylation of VE-cadherin. J Biochem. 2017;162(1):55–62. 28158602 10.1093/jb/mvx001

[32] Tremblay PL, Auger FA, Huot J. Regulation of transendothelial migration of colon cancer cells by E-selectin-mediated activation of p38 and ERK MAP kinases. Oncogene. 2006;25(50):6563–73. 16715142 10.1038/sj.onc.1209664

[33] Privratsky JR, Paddock CM, Florey O, Newman DK, Muller WA, Newman PJ. Relative contribution of PECAM-1 adhesion and signaling to the maintenance of vascular integrity. J Cell Sci. 2011;124(Pt 9):1477–85. 21486942 10.1242/jcs.082271 PMC3078814

[34] Eshaq RS, Harris NR. Loss of platelet endothelial cell adhesion Molecule-1 (PECAM-1) in the diabetic retina: role of matrix metalloproteinases. Invest Ophthalmol Vis Sci. 2019;60(2):748–60. 30793207 10.1167/iovs.18-25068 PMC6385619

[35] Cao S, Wang Y, Li J, Ling X, Zhang Y, Zhou Y, et al. Prognostic implication of the expression level of PECAM-1 in Non-small cell lung Cancer. Front Oncol. 2021;11:587744. 33828969 10.3389/fonc.2021.587744 PMC8019905

[36] Ahirwar DK, Nasser MW, Ouseph MM, Elbaz M, Cuitiño MC, Kladney RD, et al. Fibroblast-derived CXCL12 promotes breast cancer metastasis by facilitating tumor cell intravasation. Oncogene. 2018;37(32):4428–42. 29720724 10.1038/s41388-018-0263-7 PMC7063845

[37] Cen J, Feng L, Ke H, Bao L, Li LZ, Tanaka Y et al. Exosomal Thrombospondin-1 disrupts the integrity of endothelial intercellular junctions to facilitate breast Cancer cell metastasis. Cancers (Basel). 2019;11(12). 10.3390/cancers11121946 PMC6966578 31817450

[38] Benson AB, Venook AP, Al-Hawary MM, Arain MA, Chen YJ, Ciombor KK, et al. Colon cancer, version 2.2021, NCCN clinical practice guidelines in oncology. J Natl Compr Canc Netw. 2021;19(3):329–59. 33724754 10.6004/jnccn.2021.0012

[39] Shao Y, Chen T, Zheng X, Yang S, Xu K, Chen X, et al. Colorectal cancer-derived small extracellular vesicles Establish an inflammatory premetastatic niche in liver metastasis. Carcinogenesis. 2018;39(11):1368–79. 30184100 10.1093/carcin/bgy115

[40] Heijstek MW, Kranenburg O, Borel Rinkes IH. Mouse models of colorectal cancer and liver metastases. Dig Surg. 2005;22(1–2):16–25. 15838167 10.1159/000085342

[41] Franko J, Shi Q, Meyers JP, Maughan TS, Adams RA, Seymour MT, et al. Prognosis of patients with peritoneal metastatic colorectal cancer given systemic therapy: an analysis of individual patient data from prospective randomised trials from the analysis and research in cancers of the digestive system (ARCAD) database. Lancet Oncol. 2016;17(12):1709–19. 27743922 10.1016/S1470-2045(16)30500-9

[42] ATCC. American Type Culture Collection. The Global Bioresource Center 2024 [Available from: https://www.atcc.org/

[43] Plantureux L, Mège D, Crescence L, Carminita E, Robert S, Cointe S, et al. The interaction of platelets with colorectal Cancer cells inhibits tumor growth but promotes metastasis. Cancer Res. 2020;80(2):291–303. 31727628 10.1158/0008-5472.CAN-19-1181

[44] Sebastiano M, Momi S, Falcinelli E, Bury L, Hoylaerts MF, Gresele P. A novel mechanism regulating human platelet activation by MMP-2-mediated PAR1 biased signaling. Blood. 2017;129(7):883–95. 28034890 10.1182/blood-2016-06-724245

[45] Sheu JR, Fong TH, Liu CM, Shen MY, Chen TL, Chang Y, et al. Expression of matrix metalloproteinase-9 in human platelets: regulation of platelet activation in in vitro and in vivo studies. Br J Pharmacol. 2004;143(1):193–201. 15289295 10.1038/sj.bjp.0705917 PMC1575278

[46] Dashevsky O, Varon D, Brill A. Platelet-derived microparticles promote invasiveness of prostate cancer cells via upregulation of MMP-2 production. Int J Cancer. 2009;124(8):1773–7. 19101987 10.1002/ijc.24016

[47] Janowska-Wieczorek A, Marquez-Curtis LA, Wysoczynski M, Ratajczak MZ. Enhancing effect of platelet-derived microvesicles on the invasive potential of breast cancer cells. Transfusion. 2006;46(7):1199–209. 16836568 10.1111/j.1537-2995.2006.00871.x

[48] Janowska-Wieczorek A, Wysoczynski M, Kijowski J, Marquez-Curtis L, Machalinski B, Ratajczak J, et al. Microvesicles derived from activated platelets induce metastasis and angiogenesis in lung cancer. Int J Cancer. 2005;113(5):752–60. 15499615 10.1002/ijc.20657

[49] Patras L, Shaashua L, Matei I, Lyden D. Immune determinants of the pre-metastatic niche. Cancer Cell. 2023;41(3):546–72. 36917952 10.1016/j.ccell.2023.02.018 PMC10170403

[50] Coenen DM, Mastenbroek TG, Cosemans J. Platelet interaction with activated endothelium: mechanistic insights from microfluidics. Blood. 2017;130(26):2819–28. 29018081 10.1182/blood-2017-04-780825

[51] Provenzale I, Solari FA, Schönichen C, Brouns SLN, Fernández DI, Kuijpers MJE, et al. Endothelium-mediated regulation of platelet activation: involvement of multiple protein kinases. Faseb J. 2024;38(4):e23468. 38334433 10.1096/fj.202300360RR

[52] Przygodzki T, Talar M, Kassassir H, Mateuszuk L, Musial J, Watala C. Enhanced adhesion of blood platelets to intact endothelium of mesenteric vascular bed in mice with streptozotocin-induced diabetes is mediated by an up-regulated endothelial surface deposition of VWF - In vivo study. Platelets. 2018;29(5):476–85. 28745543 10.1080/09537104.2017.1332365

[53] Terrisse AD, Puech N, Allart S, Gourdy P, Xuereb JM, Payrastre B, et al. Internalization of microparticles by endothelial cells promotes platelet/endothelial cell interaction under flow. J Thromb Haemost. 2010;8(12):2810–9. 21029362 10.1111/j.1538-7836.2010.04088.x

[54] Zhang C, Wang XY, Zhang P, He TC, Han JH, Zhang R, et al. Cancer-derived Exosomal HSPC111 promotes colorectal cancer liver metastasis by reprogramming lipid metabolism in cancer-associated fibroblasts. Cell Death Dis. 2022;13(1):57. 35027547 10.1038/s41419-022-04506-4 PMC8758774

[55] Kobayashi S, Kondo N, Tomiyama T, Nakamura N, Masuda M, Matsumoto Y, et al. Intravenous injection of tumor extracellular vesicles suppresses tumor growth by reducing the regulatory T cell phenotype. Cancer Immunol Immunother. 2023;72(11):3651–64. 37597014 10.1007/s00262-023-03517-0 PMC10991856

[56] Jing B, Gai Y, Qian R, Liu Z, Zhu Z, Gao Y, et al. Hydrophobic insertion-based engineering of tumor cell-derived exosomes for SPECT/NIRF imaging of colon cancer. J Nanobiotechnol. 2021;19(1):7. 10.1186/s12951-020-00746-8 PMC7789573 33407513

[57] Ji Q, Zhou L, Sui H, Yang L, Wu X, Song Q, et al. Primary tumors release ITGBL1-rich extracellular vesicles to promote distal metastatic tumor growth through fibroblast-niche formation. Nat Commun. 2020;11(1):1211. 32139701 10.1038/s41467-020-14869-x PMC7058049

[58] Hwang WL, Lan HY, Cheng WC, Huang SC, Yang MH. Tumor stem-like cell-derived Exosomal RNAs prime neutrophils for facilitating tumorigenesis of colon cancer. J Hematol Oncol. 2019;12(1):10. 30683126 10.1186/s13045-019-0699-4 PMC6347849

[59] Huis In ‘t Veld RV, Lara P, Jager MJ, Koning RI, Ossendorp F, Cruz LJ. M1-derived extracellular vesicles enhance photodynamic therapy and promote immunological memory in preclinical models of colon cancer. J Nanobiotechnology. 2022;20(1):252. 10.1186/s12951-022-01448-z PMC9164362 35658868

