# Supplementary material for: Platelet-derived microparticles increase the interaction of colorectal cancer cells with the endothelium to promote metastatic events
Source: J Transl Med. 2025 Jul 25;23:843. doi: 10.1186/s12967-025-06858-9 (PMC12296591; doi:10.1186/s12967-025-06858-9)
Supplement: Supplementary file 1 — Supplementary Material 1 [file 12967_2025_6858_MOESM1_ESM.doc]

**Supplementary Methods**

**Cell culture**

Human colorectal adenocarcinoma cell lines with different phenotypic migratory potentials, including the CRC cell lines HT29 (epithelial, CMS3), SW480 (mesenchymal, CMS4), and SW620 (strongly mesenchymal, CMS4) as well as the endothelial HMEC-1 cell line (immortalised dermal microvascular endothelial cells) (ATCC CRL-3243™) were purchased from the American Type Culture Collection (ATCC, Manassas, VA, USA). SW480 and SW620 originate from the same patient - primary adenocarcinoma of the colon (SW480) and metastatic lesion in lymph node taken one year later (SW620). HT29 cells were cultured in McCoy’s 5A medium (Thermo Fisher Scientific, Waltham, MA, USA), SW480 and SW620 cells were cultured in RPMI 1640 medium (with the ATCC modification, Thermo Fisher Scientific). The HMEC-1 cell line was cultured in MCDB131 medium (supplemented with the following growth factors: 10 ng/mL epidermal growth factor, 1 µg/mL hydrocortisone, and 10 mM glutamine ). All media for culturing the cells were supplemented with fetal bovine serum (final conc. 10% FBS; Sigma–Aldrich), penicillin/streptomycin (final conc. 100 U/ml/100 µg/ml, Thermo Fisher Scientific) and primocin (final conc. 0.1 mg/ml, InvivoGen, San Diego, CA, USA). Cells were cultured at 37 ˚C in 5% CO_2_ and 95% humidity. Cells were routinely tested for mycoplasma (PlasmoTest, InvivoGen). Unless otherwise stated, all of the experiments were performed in FBS-free media (experimental media). For the experimental studies, CRC cells between the 10th and 20th passages and HMEC-1 cells between the 3rd and 9th passages were used.

**Isolation and characteristics of platelet-derived microparticles (PMP)**

PMP were obtained from platelet concentrates (outdated as far as patients treatment is concerned) purchased from the Regional Centre of Blood Donation and Blood Treatment in Łódź in accordance with applicable law. In accordance with applicable law, the purchase does not require the approval of ethics committee. First, the platelets were separated from the plasma via centrifugation at 200 × g for 20 min at RT. After two washes with Tyrode’s buffer, the platelets were stimulated with 2 U/mL thrombin and 2.5 mM CaCl_2_ for 20 min on a rotary shaker. PMP were obtained after centrifugation of stimulated platelets for 1,500 × g for 20 min at RT followed by ultracentrifugation of the resulting suspension at 100,000 × g for 2 h at 4 °C.

Protein concentration of PMP was determined using the BCA Protein Assay Kit (Pierce, Thermo Fisher Scientific) according to the manufacturer’s instructions. Protein content in conditioned medium was assayed using NanoDrop™ 1000 Spectrophotometer (Thermo Fisher Scientific).

PMP size distribution and quantification were analysed by NTA (Nanoparticle Tracking Analysis) using NanoSight NS300 System (Malvern Panalytical Ltd., Malvern, UK) by a courtesy of the representative of company (A.P. Instruments, Warsaw, Poland).

**Preparation of conditioned media after CRC cell stimulation with PMP**

CRC cells were seeded in 24-well plates for 48 h. At 80-90% confluence, the medium was replaced with experimental medium, and PMP were added (50 µg of PMP/10^6^ cells for a final concentration of 100 µg/mL) for 24 h. Subsequently , the conditioned medium (obtained from PMP-stimulated CRC cells and from nonstimulated CRC cells [as a control]) was collected, and centrifuged at 180 × g for 5 min at RT to remove cellular debris; afterwards the supernatant was collected and frozen at -20 °C until use.

**CRC cell adhesion to the endothelial cell layer**

HMEC-1 cells (4 × 10^5^) were seeded in 24-well plates for 48 h. At 80-90% confluence, full medium was changed to medium without EGF and hydrocortisone for 16 h before starting the experiment. Then PMP in experimental medium (50 µg of PMP /10^6^ HMEC-1 cells, final concertation of 100 µg/mL) were added and incubated for 4 h. After washing of HMEC-1 with PBS, CRC cells (2 × 10^5^ in medium with 1% FBS) previously labelled with CellTracker™ Green CMFDA dye according to the manufacturer instruction, were added onto HMEC-1 layer. CRC cells were left for 3 h to adhere to the endothelial cell layer. In another set of experiments, CRC cells, labelled with CellTracker™ Green CMFDA dye, before loading on the endothelial cell layer, were incubated with PMP (50 µg of PMP /10^6^ CRC cells, final protein concentration 100 µg/ml) for 4 h. The third tested model was the incubation both, HMEC-1 cells and CRC cells with PMP before the loading CRC cells on the endothelial cell layer for 3 h. In all of three tested models, for inhibition of PMP incorporation, we used dynamine inhibitor, Dynasore (final concertation of 50 µM) (Abcam, Cambridge, Great Britain) or DMSO (final concertation of 0.1 %, control) for 4 h in appropriate medium not supplemented with FBS. After washing with PBS, the fluorescent CRC cells adhered to HMEC-1 cells were count using a Nikon inverted fluorescence microscope (Eclipse E600; Nikon, Tokyo, Japan) at 200× magnification.

**Assessment of the integrity of the HMEC-1 monolayer after conditioned medium or PMP treatment**

HMEC-1 cells (4x10^5^/well) were seeded in 24-well plates for 48 h. At 80–90% confluence, full medium was changed to medium without EGF and hydrocortisone for 16 h before starting the experiment. Next, the cells were incubated with CellTracker™ Green CMFDA due (Thermo Fisher Scientific) (final concertation of 5 µM) in medium containing 1% FBS for 30 min and washed with PBS in order to remove unbound dye. The conditioned media of previously PMP-treated CRC cells (final concertation of . 100 µg/ml) or PMP alone (final concertation of 100 µg/ml) were added to HMEC-1 for 4 h. After washing with PBS, the fluorescence was evaluated using a Nikon inverted microscope (Eclipse E600; Nikon, Tokyo, Japan) at 200× magnification. The areas covered with fluorescently labelled HMEC-1 cells were calculated using ImageJ Software (Fiji). A quantitative barrier assay was used as previously described in (14) (with some modifications). Briefly, HMEC-1 cells (4 × 10^5^) were seeded in full medium on 0.4 μm transwell inserts in the 24-well plate for 3 h, after which the cells were treated with conditioned media or PMP as described above. Dextran conjugated with fluorescein isothiocyanate (FITC) (1 µg/mL) was added to the upper chamber and the amount of diffused Dextran-FITC in the lower chamber that passed through the endothelial barrier was measured by fluorescence intensity at 488 nm via a fluorescence microplate reader.

**mRNA isolation and real‑time PCR analysis of endothelial gap and tight junction protein expression**

Total RNA was isolated by using the Monarch Total RNA Miniprep Kit (New England Biolabs, MA, USA) according to the manufacturer’s instructions. A total of 0.5 μg of the isolated total RNA was reverse transcribed via a High-Capacity cDNA Reverse Transcription Kit (Applied Biosystems, Waltham, MA, USA) according to the manufacturer’s instructions. Human *PECAM1, ZO1, CDH5* and *GAPDH* genes were analysed via real-time polymerase chain reaction (PCR) by using Fast-Start Essential DNA Green Master Mix (Roche, Basel, Switzerland) with specific primers (Table S2). Amplification was performed on a Roche LightCycler 96. A standard PCR experiment was the following: preincubation at 95 °C for 600 sec, followed by 45 cycles of 95 °C for 10 sec and 60 °C for 10 sec, 72 °C for 10 sec, and finishing with one cycle of 95 °C for 10 sec, 65 °C for 60 sec, 97 °C for 1 sec. *GADPH* transcripts were used as internal controls. The amount of target in the various samples was calculated using the 2^(−ΔCt) relative quantification method with DataAssist v.3.01.

**Determination of the presence of tight junction proteins in HMEC-1 cells**

For flow cytometry, HMEC-1 (4x10^5^/well) cells were seeded in the 24-well plates for 48 h. At 80–90% confluence, full medium was changed by the medium without EGF and hydrocortisone for 16 h before starting the experiment. The conditioned media of previously PMP-treated CRC cells (final concentration of 100 µg/ml) or PMP alone (final concentration of 100 µg/ml) were added to HMEC-1 for 24 h. Accutase (Sigma Aldrich) was used instead of routinely used trypsin for cells detaching since accutase rather than trypsin is recommended for flow cytometric measurements of surface antigens. For labelling with antibodies, 10^6^ cells per milliliter were applied. Cells were incubated with 2 µg of rabbit polyclonal anti-human PECAM-1 antibody (Santa Cruz Biotechnology) or rabbit monoclonal anti-human ZO-1 antibody (D7D12, Abcam) for 1h at RT followed by incubation with secondary goat anti-rabbit antibody conjugated with Alexa Fluor488 (Invitrogen) for 1 h at RT. Samples were washed after each labelling in PBS containing 1% BSA in order to remove resting and unbound antibodies and fixed with 1% Cellfix (BD Biosciences) for 1 h at RT. Flow cytometric measurements and analysis was performed as described above. Results were presented as MFI values for PECAM-1 or ZO-1 positive cells. The specific fluorescence was evaluated after subtracting of the MFI values for binding of secondary antibodies.

For confocal microscopy, HMEC-1 cells were seeded in 8-well poly-lysine coated glass chamber slides, washed in PBS and incubated with the conditioned media of previously PMP-treated CRC cells (at final protein concentration 100 µg/ml) or PMPs alone (final concentration of 100 µg/ml) in the medium with 0% FBS for 24 h. After washing in PBS, slides were fixed with 3% PFA at RT for 10 min followed by incubation with 0.1% Triton X-100 for 20 min at RT for cell permeabilisation. Excessive amount of PFA and Triton X-100 were removed by intermediate washing steps in PBS. Then the particular cell components were labeled after addition of following dyes: Alexa-Fluor 594-conjugated Wheat germ agglutinin (5 µg/ml, plasma membrane labeling dye) and Hoechst 33342 (5 µg/ml cell-permeant nuclear dye) while PECAM and ZO-1 proteins were identified using specific primary antibodies and secondary antibodies mentioned above. After removal the unbound antibodies and dyes by washing in PBS, cells on slides were visualized using confocal microscope (Nikon D-Eclispe C1) analyzed with EZ-C1 version 3.6 software.

For immunoblotting detection of total PECAM-1 and ZO-1 proteins, HMEC-1 cells were incubated for 24h with PMP as described above and lysed using RIPA buffer (Sigma Aldrich). The lysates (20 µg of protein) were then separated on an SDS‒PAGE 10% polyacrylamide gel, transferred to a nitrocellulose membrane and incubated with mouse anti-human antibodies against PECAM-1 (89C2; Cell Signaling, #3528) or rabbit anti-human antibodies against ZO-1 (Proteintech, 21773-1-AP), followed by incubation with secondary horse anti-mouse IgG (Cell Signaling, #7076) or goat anti-rabbit IgG (Invitrogen, #31460) conjugated with horseradish peroxidase (HRP).

**Transendothelial migration assay**

Transendothelial migration assay was performed based on the published protocol (Cen et al., 2019) with minor modifications. Briefly, HMEC-1 cells (4 × 10^5^) in full medium were seeded into the inserts in Transwell System (Nunc™ Polycarbonate Cell Culture Inserts in Multi-Well Plates, 8 μm, Thermo Fisher Scientific). After 3 h, PMP (100 µg/ml) or equal volume of PBS (control) in experimental medium were added and the transwell plates were incubated for 4 h. Meanwhile, CRC cells (2 × 10^5^) were incubated with CellTracker™ Green CMFDA dye (Thermo Fisher Scientific) for 30 min (final concentration of 5 µM) in medium containing 1% FBS and after removing the unbound dye (centrifugation 180×g, 5 min, RT), the cells were suspended in medium containing 1% FBS. Then, transwells covered by HMEC-1 cells were washed with PBS and CRC cells were loaded on HMEC-1 layer. Full medium of each CRC cell line was added to into the lower chamber as chemoattractant. The migration of CRC through HMEC-1 layer was tested after 24 h. After washing with PBS, the cells on the top side of the insert transwell membrane were scraped off and the number of cells that passed through to the filter was evaluated on the undersides of filters by Nikon inverted microscope (Eclipse E600; Nikon, Tokyo, Japan) at 200× magnification. Cell migration across the membrane was quantified randomly in five fields at different locations for each membrane.

**Mouse model of colorectal cancer metastasis** All experiments were performed in accordance with the guidelines formulated by the European Community for the Use of Experimental Animals (L358-86/609/EEC) and the Guide for the Care and Use of Laboratory Animals published by the US National Institute of Health (NIH Publication No. 85–23, revised 1985). All procedures were approved by Local Ethics Committee on Animal Experiments at the Medical University in Lodz (approval number: 38/Ł 241/2022). Balb/c nude mice (female, age 6-8 weeks) were purchased from sales distributor/representant of Charles River Laboratories - Animalab. Allocation to experimental groups was based on simple randomization. During the experiments, the animals were housed under sterile conditions in special cages connected to the separate sterile air vents with free access to sterilized water and special chow for immunodeficient rodents (Altromin Maintenance Diet). On the day of the experiment, mice were anaesthetized by the intraperitoneally injection of the mixture of ketamine (100 mg/kg b/w/) and xylazine (10 mg/kg b.w.). Additionally, analgesic drug, butorphanol (2 mg/kg b.w.), was applied during anesthesia and later, 4 times at 8-12h intervals. The spleen was surgically exposed and the 15 µl of CRC cells suspension (1.5×10^6^ of HT29, SW480 or SW620 cells in Hank’s Balanced Salt Solution) (6 mice per CRC cell line) or HBSS alone (control) (4 mice) was injected in the middle part of the spleen with Hamiltonian syringe. We did not observe any behavioral changes in the animals (such as apathy or hyperactivity, decreased or increased appetite) that would indicate the occurrence of inflammation directly after cell injection. Spleen was then placed into abdominal cavity and skin and muscle layers were sutured. Mice were intravenously injected with 100 µl of PMP (at the dose of 20 µg in PBS) or with PBS (control) every 7 days for the next 5 weeks starting on the seventh day after intrasplenic injection. Mice weight was monitored every 7 day. During section at the end of the experiment (on the 35th day), mice were anaesthetized as described above, blood was terminally collected on the EDTA, part of the blood was intended for determination of platelet activation and reactivity while part of the blood was centrifuged at 1,000×g for 15 min RT and the resulted plasma was frozen at -80 °C for further analyses. Liver, spleen, colon and lungs were surgically excised and macroscopically analyzed by veterinarian to detect metastases. Then resected organs were fixed in 10% buffered formalin for further histopathological and immunohistochemical examination.

**Histopathological examination** Formalin-fixed organs were automatically processed and embedded in paraffin. The 4 μm-thick sections were cut and mounted on SuperFrost® Plus slides, and then processed for routine staining with Mayer’s hematoxylin and eosin to microscopically detect metastases and inflammation. Additionally, immunohistochemistry was performed using antibodies against CDX2 (Clone DAK-CDX2), CK20 (Clone Ks20.8), and CD41 (polyclonal) with the FLEX system (Dako, Agilent) on the Autostainer Link 48 (Dako, Agilent). Imaging was performed using the AxioLab 5 microscope (Carl Zeiss, Oberkochen, Germany) with a standard Axiocam 208 color camera (Carl Zeiss, Oberkochen, Germany). Image analysis was conducted semi-automatically using AxioVision 4.8 software and ZEN (blue edition) 3.3 software (Carl Zeiss, Oberkochen, Germany).

Inflammatory changes were assessed using a widely used grading system that evaluates portal/periportal and lobular inflammatory activity. To reflect the context of this murine model, we applied a simplified, semi-quantitative 0–3 scale focused on inflammatory activity, based on the predominant portal or lobular involvement.

**Flow cytometric determination of platelet activation**

Circulating platelet activation, and platelet reactivity in response to thrombin (at the final concentration of 0.25 U/ml) were evaluated on the basis of the measured expressions of specific surface membrane antigens CD62P (P-selectin) and activated αIIbβ3 complex using specific rat anti-mouse antibodies, conjugated with PE (Emfret Analytics). Platelets were gated on the basis of the binding of αIIbβ3 (non-activated complex) antibodies conjugated with FITC. Flow cytometric measurements were performed using LSRII instrument (BD Biosciences, San Jose, CA). 10,000 cells were analyzed per sample. All data were processed using FACS/Diva ver. 6.0 software (BD Biosciences, San Jose, CA). The percent fractions of specific fluorescence-positive platelets were evaluated after subtracting the binding of nonspecific isotype mouse IgG1. Results were presented as the percent fractions for CD62P- or activated αIIbβ3 -positive platelets within gated population of platelets.

**Quantification of cytokines in mouse plasma**

For quantification of six murine cytokines (IL-12p70, IL-10, IL-6, MCP-1, IFN-γ, TNF-𝛼) in plasma, bead-based multiplex analysis was performed using a commercial BD Cytometric Bead Array (CBA) Mouse Inflammation Kit, according to the manufacturer’s protocol. Bead fluorescence was acquired with a BD LSR II and analyzed in FCAP Array Software (BD Bioscience). Target concentration was quantified against a known standard supplied by the manufacturer at a given concentration. The mean fluorescence intensity of each dilution series of the standard aids in generating 10-point dilution standard curves that allow the absolute quantification of each target investigated in the cell culture supernatant. Any value below the limits of detection was given zero for that cytokine.

**Enzyme-linked immunosorbent assay for MMP-2 and MMP-9**

Concentrations of total MMP-2 and human MMP-9 in mice plasma were determined using Total MMP-2 Quantikine ELISA Kit and Quantikine Human MMP-9 ELISA Kit, according to the manufacturers’ protocols. Before assay, collected plasma was additionally centrifuged at 10,000 × g for 10 min at 4°C to complete platelet removal and to obtain platelet-free plasma. Plasma dilution was optimized in pilot studies and final 20-fold dilution for MMP-2 assay and 10-fold dilution for MMP-9 assay were applied. Concentration of MMP-2 and MMP-9 in plasma were evaluated according to appropriate standards of tested metalloproteases.

**Supplementary Figures and Tables**

**
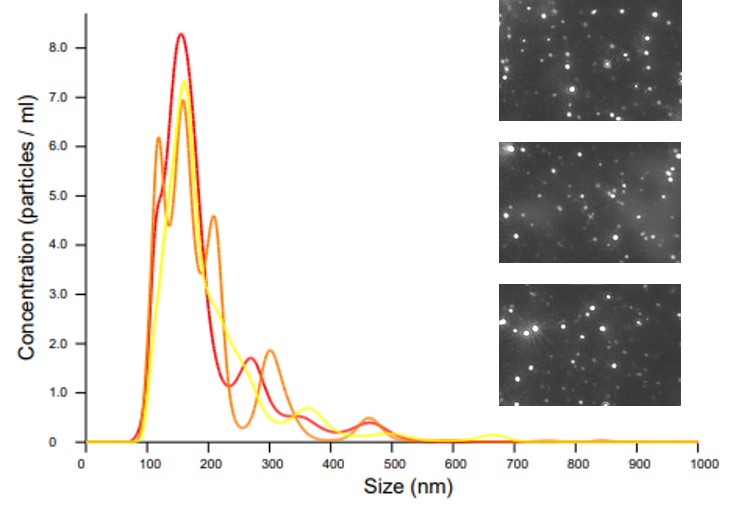
**

**Fig. S1. Size characteristics of PMPs.** Representative graph from Nanoparticle Tracking Analysis (NTA) of PMPs isolated from thrombin-stimulated platelets.

NTA showed similar sizes of PMP isolated from different platelet concentrates (213.8 ± 4.4 nm, mean ± SE, N=2).


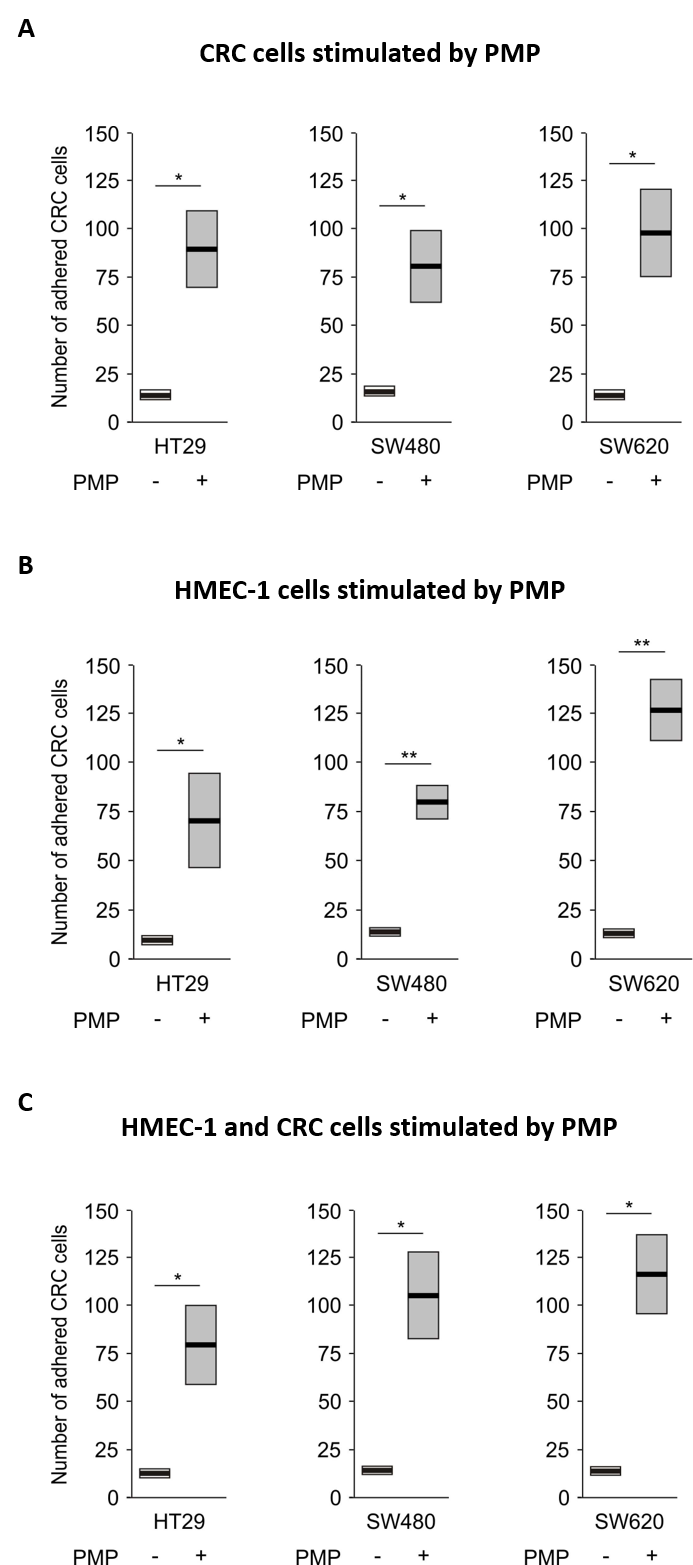


**Fig. S2. The effect of PMP on the adhesion of CRC cells to HMEC-1 monolayer.** CRC cells (A), HMEC-1 cells (B) or both (C) were previously preincubated with PMP (50 µg of PMPs/ 10^6^ of cells, final concentration of 100 µg/ml) for 4 h. Data are presented as the means (vertical lines) and standard errors (boxes) of number of adhered, fluorescently labelled CRC cells after 3 h incubation. Statistics were calculated with a parametric Student’s t test: ** P<0.01, * P < 0.05, N=3.

**
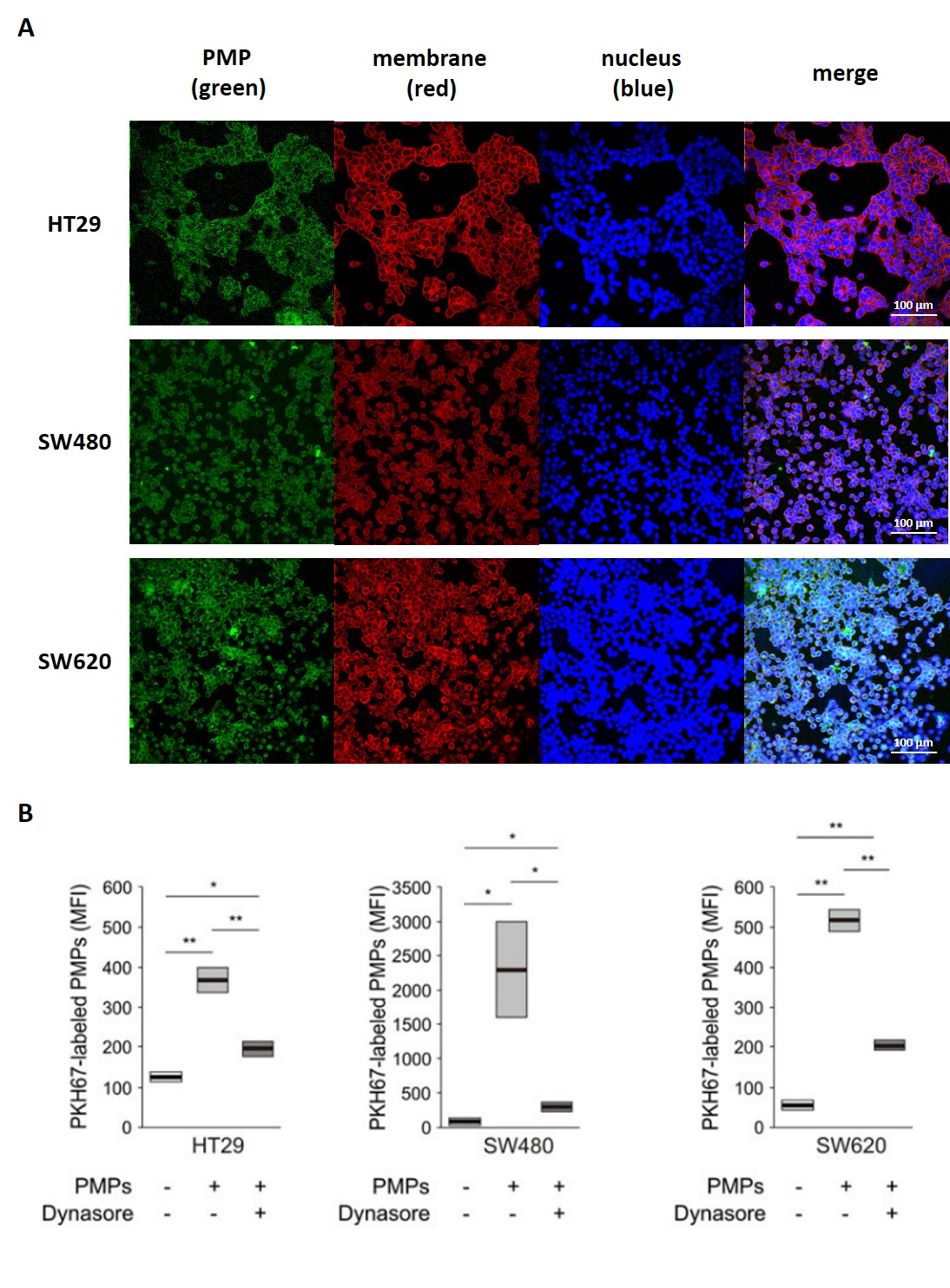
**

**Fig. S3. The internalization of PMPs by CRC cell lines.** A – Representative confocal microscopy images of PKH67-labelled PMPs uptake (green) by CRC cells (50 µg of PMPs/ 10^6^ of cells, final concentration of 100 µg/ml) after 4 h incubation at 37°C in a humidified atmosphere with 5% CO_2_. (400x magnification). Samples were stained with Hoechst 33342 (blue) for nuclei and Alexa Fluor 594 (red) wheat germ agglutinin for cell membrane. B – Flow cytometry analysis of PKH67-labelled PMPs uptake by CRC cells (50 µg of PMPs/ 10^6^ of cells, final concentration of 100 µg/ml) after 4 h. Incorporation of PMPs was blocked by dynamin inhibitor, Dynasore (at the final concentration of 100 µM). Data are presented as the means (vertical lines) and standard errors (boxes) of MFI values for green fluorescence (derived from PKH67-labeled PMPs) in CRC cells. Statistics were calculated with a parametric Student’s t test or nonparametric Mann-Whitney test: *P<0.05, **P<0.01. N= 3. Related to Figure 3.


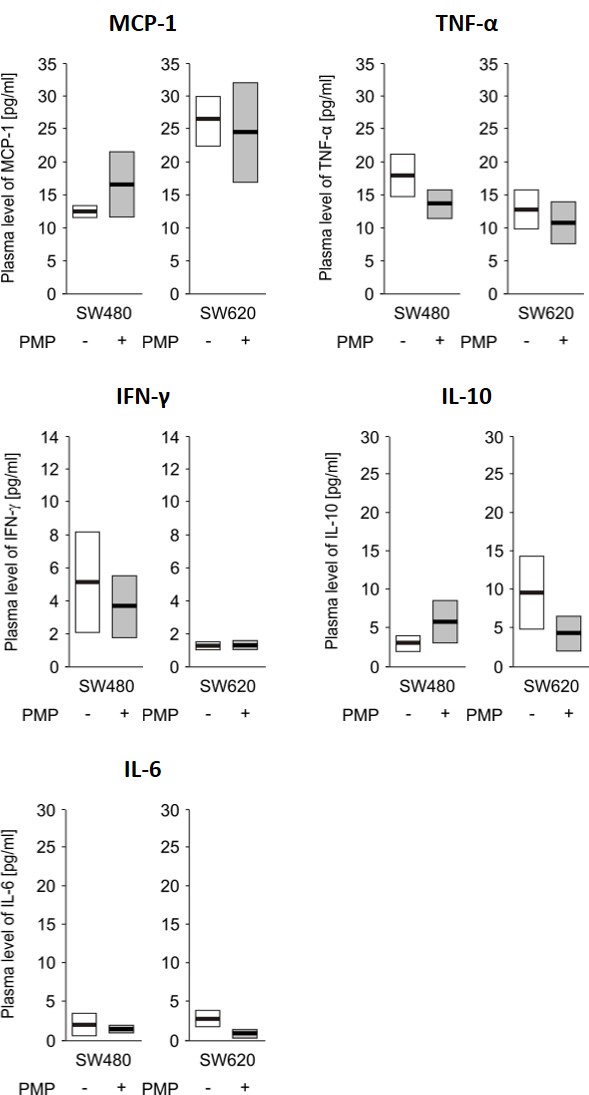


**Fig. S4. The effect of PMPs on the level of plasma inflammatory markers in CRC in vivo model.** Level of MCP-1, TNF-α, IFN-γ, IL-10 and IL-6 in plasma from CRC mouse model intravenously injected with PMPs. Quantified data are presented as mean (vertical line) and standard error (box).

**
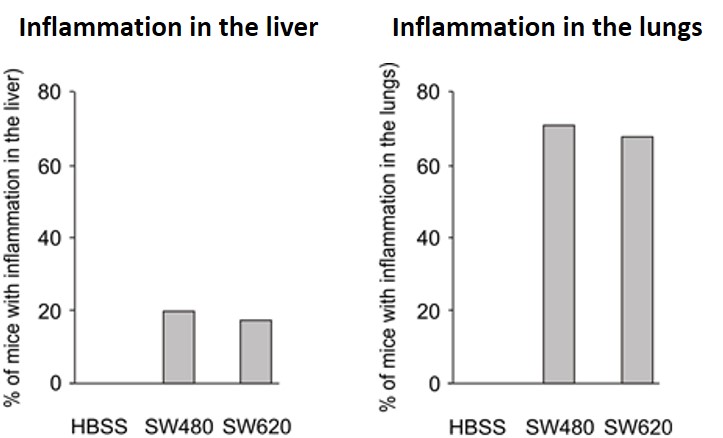
**

**Fig. S5**. **Inflammation in liver and lungs in mice model of colorectal cancer.** Results are presented as percentage of mice with inflammation detected in liver (left) and lungs (right) in whole group of animals injected with HBSS (control), SW480 and SW620 cells.

**Table S1. Incidences of pathological abnormalities other than inflammation and liver metastases in mice with SW480 or SW620 cells intrasplenic injection followed by injections of PMPs.**

| Group | Cell injection day 0 | PMPs or PBS injections every 7 days | Pathological abnormalities other than liver metastases | | | | | |
| --- | --- | --- | --- | --- | --- | --- | --- | --- |
|  |  |  | colon thicker walls | enlarged lymph nodes | Metastases in the net | Metastases in the peritoneum | tumor of the serosa of the large intestine | Abnormal cutting line |
| 1 | HBSS | PBS | 0/6 | 0/6 | 0/6 | 0/6 | 0/6 | 0/6 |
| 2 | SW480 | PBS | 2/5 | 4/5 | 1/5 | 0/5 | 0/6 | 3/5 |
| 3 | SW480 | PMPs | 1/5 | 5/5 | 0/5 | 0/5 | 0/6 | 2/5 |
| 4 | SW620 | PBS | 0/6 | 6/6 | 3/6 | 0/6 | 0/6 | 6/6 |
| 5 | SW620 | PMPs | 2/6 | 5/6 | 0/6 | 1/6 | 1/6 | 3/6 |

**Table S2. List of primers used in this study.**

| Gene | sequence of forward primer 5-3 | sequence of reverse primer 5-3 | Amplicon size | Accession number |
| --- | --- | --- | --- | --- |
| *GADPH* | TTGCCCTCAACGACCACTTT | TCCTCTTGTGCTCTTGCTGG | 146 | NM_001357943.2 |
| *PECAM1* | GTCAAGCCTCAGCACCAGAT | CACCTGGTACTCTGCAGTGG | 177 | XM_017024741.2 |
| *ZO1/TJP1* | CAACATACAGTGACGCTTCACA | CACTATTGACGTTTCCCCACTC | 105 | XM_047432991.1 |
| *CDH5* | ATGTAGGCAAGATCAAGTCAAG | CCTCTCAATGGCGAACAC | 122 | XM_047433471.1 |

*GADPH*; Homo sapiens glyceraldehyde-3-phosphate dehydrogenase, *PECAM1*; Homo sapiens platelet and endothelial cell adhesion molecule 1, *ZO1*; Homo sapiens tight junction protein 1 (*TJP1*), *CDH5*; Homo sapiens Vascular endothelial cadherin
